# High-CD14-expressing urothelial cancer cells foster a neutrophil-rich tumor microenvironment that increases the risk of radiation-promoted distant metastasis

**DOI:** 10.1186/s12929-025-01201-2

**Published:** 2026-01-04

**Authors:** Yun Chiang, Yu-Chieh Tsai, Chung-Chieh Wang, Fu-Jen Hsueh, Chao-Yuan Huang, Chung-Hsin Chen, Yeong-Shiau Pu, Shih-Chieh Chueh, Xavier Cheng-Hong Tsai, Jason Chia-Hsien Cheng

**Affiliations:** 1 Graduate Institute of Oncology, National Taiwan University College of Medicine, Taipei, Taiwan; 2 Department of Radiation Oncology, National Taiwan University Cancer Center, Taipei, Taiwan; 3 Division of Medical Oncology, Department of Oncology, National Taiwan University Hospital, Taipei, Taiwan; 4 Department of Pathology, National Taiwan University Hospital, Taipei, Taiwan; 5 Department of Urology, National Taiwan University Hospital, Taipei, Taiwan; 6 Division of Hematology, Department of Internal Medicine, National Taiwan University Hospital, Taipei, Taiwan; 7 Division of Radiation Oncology, Department of Oncology, National Taiwan University Hospital, Taipei, Taiwan

**Keywords:** Metastasis, Neutrophil, Radiotherapy, Tumor microenvironment, CD14

## Abstract

**Background:**

Radiation (RT)-promoted distant metastasis (DM) is an underrecognized complication that can compromise the therapeutic efficacy of local RT. This study aimed to identify tumor microenvironment (TME) traits that predispose to RT-promoted DM and provide mechanistic insights for potential therapeutic intervention.

**Methods:**

We performed NanoString analysis on tumor samples from urothelial cancer patients to compare the TME profiles of those with and without RT-promoted DM. To complement clinical findings, we conducted RNA sequencing (RNAseq) of murine bladder cancer cell lines, MB49 (with RT-promoted DM) and MBT2 (without), followed by in vivo ectopic tumor modeling, flow cytometry of immune cell infiltrates, and cytokine array profiling.

**Results:**

NanoString analysis revealed a significant enrichment of C-X-C motif receptor 2 (CXCR2)-expressing neutrophils in the TME of patients with RT-promoted DM. These tumors also exhibited nuclear factor kappa B (NFκB) activation and increased secretion of neutrophil-recruiting chemokines. RNAseq and cytokine profiling identified CD14 expression in tumor cells as a key upstream regulator of neutrophilic TME via NFκB signaling. The use of antagonists to block neutrophils and inhibit CD14 expression in cancer cells, which reduces the secretion of neutrophil-recruiting chemokines, effectively mitigated RT-promoted DM in both the MB49 and LLC mouse models.

**Conclusions:**

CD14 expression in tumor cells plays a pivotal role in shaping a neutrophil-enriched TME, which increases the susceptibility to RT-promoted DM. CD14 represents a potential predictive biomarker and therapeutic target for mitigating this adverse outcome.

**Supplementary Information:**

The online version contains supplementary material available at 10.1186/s12929-025-01201-2.

## Background

Radiotherapy stands as a cornerstone in cancer care, with more than half of cancer patients receiving it as an integral part of their treatment regimen [1]. However, the increased migratory ability of radiation (RT)-resistant cancer cells may unintentionally promote out-of-field distant metastases, a phenomenon sometimes referred to as “badscopal effect” [2, 3]. RT-promoted metastasis is a relatively uncommon phenomenon and is not frequently observed across most tumor types. Nevertheless, there have been documented instances in both preclinical and clinical studies where such metastases have been observed [1, 4].

Our previous preclinical research revealed that subablative RT stimulated bladder cancer cells to secrete C–C motif chemokine receptor 2 (CCL2) [5], which subsequently attracted C–C motif chemokine receptor 2 (CCR2) + myeloid cells and C–C motif chemokine receptor 4 (CCR4) + regulatory T cells (Tregs) to the irradiated tumor microenvironment (TME) [5, 6]. This collective cellular response contributed to increased tumor invasion and lung metastases (LM) [6]. However, in line with the principles of personalized medicine and the idea that prevention is better than a cure, identifying the characteristics of the pre-RT TME that increase the risk of RT-promoted distant metastasis (DM) would allow for more effective treatment planning. By recognizing high-risk patients before RT, we could adjust treatment strategies to minimize this risk and avoid potentially harmful drug combinations with RT.

Given the substantial evidence highlighting the role of immune cells, such as macrophages, neutrophils, myeloid-derived suppressor cells, dendritic cells, and Tregs, in shaping an immunosuppressive TME [7], the infiltration levels of certain immune cells may imply valuable insights. Notably, neutrophils, the most abundant leukocytes in the blood, have gained increasing recognition for their emerging roles in metastasis [8, 9]. Moreover, the bidirectional communication between cancer cells and the TME is critical for tumor progression and invasion [10]. Therefore, it is equally important to explore the intrinsic markers of tumor cells that may affect the recruitment of specific immune cells.

To the best of our knowledge, this is the first study to identify microenvironmental biomarkers derived from both immune and cancer cells that indicate predisposition to RT-promoted DM. Understanding the specific features of the TME that contribute to this risk will help select patients most likely to benefit from local RT, reduce the risk of RT-promoted DM, and guide the use of targeted medications to overcome that effect.

## Methods

### Cell lines and cultures

The murine MB49 and MBT2 bladder cancer cell lines, as well as a Lewis lung carcinoma (LLC) cell line, were obtained and cultured as previously described [5, 6, 11, 12]. The murine Hepa 1–6 liver cancer cell line was obtained from American Type Culture Collection (Manassas, VA, USA), and the murine H22 liver cell line was acquired from Cell Lines Service (Eppelheim, Germany). Hepa 1–6 cells were maintained in RPMI-1640 medium supplemented with 10% fetal bovine serum (FBS), and H22 cells were cultured in high-glucose DMEM supplemented with 10% FBS. All the cell lines were cultured at 37 °C in a humidified atmosphere of 5% CO_2_ and 95% air.

### Mice

Male C57BL/6 (B6) mice, aged 5–6 weeks, were obtained from the National Taiwan University Animal Center (Taipei, Taiwan), and female C3H/H3N (C3H) mice of the same age were obtained from the National Laboratory Animal Center (Taipei, Taiwan). All experimental procedures involving these mice were conducted in accordance with protocols approved by the National Taiwan University Institutional Animal Care and Use Committee.

### In vivo* ectopic mouse model*

Ectopic tumors were established by subcutaneously injecting MB49 cells (1 × 10^6^), LLC cells (1 × 10^6^), Hepa 1–6 cells (8 × 10^6^), or H22 cells (4 × 10^6^) into the right hind limbs of B6 mice, and MBT2 cells (2 × 10^6^) into the right hind limbs of C3H mice. As the tumors developed, the mice were randomly assigned to different treatment groups. For the RT groups, thigh tumors were irradiated between 7 and 14 days after implantation. For the RT experiments with MB49, MBT2, Hepa 1–6, and H22 tumors, three 7.5-Gy fractions were administered on days 1 to 3, whereas with LLC tumors, five 10-Gy fractions were administered on days 1 to 5. RT was delivered via a small animal X-ray irradiator (X-RAD SmART [Small Animal RadioTherapy], PRECISION X-ray Irradiation, Madison, CT, USA), a microimage-guided radiotherapy (IGRT) platform that utilizes a cone beam computed tomography (CBCT) imaging system capable of delivering preclinical multibeam and multitarget 360-degree radiation, as previously described [5, 6].

### Reagents

Neutrophil depletion was implemented via the use of either a Ly6G antagonist (InVivoPlus anti-mouse Ly6G, Bio X Cell, Lebanon, NH, USA) or a CXCR2 antagonist (Reparixin, Cayman, Ann Arbor, MI, USA). For the in vivo experiments with MB49 and LLC cells, the mice received intraperitoneal injections of 0.2 mg of InVivoPlus anti-mouse Ly6G or 20 mg/kg Reparixin. Injections were administered once every three days for a total of four treatments. To inhibit CD14 expression, MB49 or LLC cells were treated with a CD14 antagonist (10 μg/ml; purified NA/LE rat anti-mouse CD14, BD Pharmingen, Franklin Lakes, NJ, USA) for 24 h. In addition, TPCA-1 (MedChemExpress, Monmouth Junction, NJ, USA), an inhibitor targeting CXCL1 signaling, was administered intraperitoneally at 20 mg/kg for in vivo studies. For in vitro experiments, cells were treated with Resatorvid (TAK-242; MedChemExpress, Monmouth Junction, NJ, USA), a TLR4 pathway inhibitor, at 10 ng/ml.

### Evaluation and interpretation of immunohistochemistry

Mice from each group were sacrificed between days 21 and 27. Tumor tissues were fixed in 10% neutral buffered formalin, processed for immunohistochemical (IHC) staining, embedded in paraffin blocks, and sectioned (10 μm thick). The sections were then incubated with primary antibodies against CXCR2 (Bioss Inc., Woburn, MA, USA; 1:200). For patient tumor samples, tissues were retrieved from paraffin blocks and incubated with an anti-CXCR2 primary antibody (Bioss Inc., Woburn, MA, USA; 1:400). IHC staining was performed using the Ventana Benchmark XT (Ventana Medical System, Tucson, AZ, USA) following the manufacturer’s protocol. Images were digitally captured on a DS-Ri2 microscope camera (Nikon, Japan). Cells with positive staining were counted in five random 400 × fields, and the average cell counts were compared across groups. Tumors with CXCR2 staining in ≥ 5% of tumor cells were defined as positive, whereas those with < 5% positive cells were considered negative. For double IHC staining, serial 4 μm paraffin sections were incubated with anti-Ly6G (clone 1A8, 1:50, BioLegend, USA) and anti-CD11b (clone EPR1344, 1:400, Abcam, UK) using the Ventana Benchmark XT platform. Labeling was visualized with the Ultraview DAB and Universal Alkaline Phosphatase Red detection kits according to the manufacturer’s instructions, and all sections were counterstained with hematoxylin. Each staining was performed on tumor tissues from at least three independent mice per group, and quantitative comparisons between groups were analyzed using the Mann–Whitney *U* test.

### Enzyme-linked immunosorbent assay (ELISA)

CXCL1 and CD14 concentrations were measured using a commercially available mouse protein sandwich enzyme immunoassay kit with two mouse monoclonal anti-human antibodies (R&D Systems, Minneapolis, MN, USA). Medium samples from culture plates, along with serum samples obtained by blood collection from submandibular vein at baseline, post-RT day 7, and post-RT day 14, as well as standards, were incubated in microplate wells precoated with the primary mouse monoclonal anti-human biomarker antibody. After washing, a secondary peroxidase (HRP)-conjugated anti-human biomarker antibody was added for subsequent incubation. The reaction between HRP and the substrate (hydrogen peroxide and tetramethylbenzidine) resulted in a color change, with intensities measured at an absorbance of 450 nm using a microplate reader. Serum biomarker concentrations were calculated on the basis of a standard curve. Each sample was assayed in duplicate, and data were analyzed using the Mann–Whitney *U* test for two-group comparisons. Results are presented as median with interquartile range (IQR).

### Reverse transcription‒polymerase chain reaction

The cDNAs for specific genes were cloned and amplified via PCR with the following primers: GAPDH (sense 5′- CTTTGTCAAGCTCATTTCCTGG -3′ and antisense 5′- TCTTGCTCAGTGTCCTTGC -3′), mouse CD14 (sense 5′- CAGAGAACACCACCGCTGTA -3′ and antisense 5′- GGACCAATCTGGCTTCGGAT -3′), mouse CXCL1 (sense 5′- ACTCAAGAATGGTCGCGAGG -3′ and antisense 5′- GTGCCATCAGAGCAGTCTGT -3′), mouse CXCL2 (sense 5′- AGGGCGGTCAAAAAGTTTGC -3′ and antisense 5′- CGAGGCACATCAGGTACGAT -3′), mouse CXCL3 (sense 5′- CCAACGGTGTCTGGATGTGT -3′ and antisense 5′- CAGCCAAGGAATACTGCCTCA -3′), mouse p50 (sense 5′- CCGGTGTCCTTTCTAGCCAT -3′ and antisense 5′- TGTAAAATGCATAAAACGGGGAA -3′), mouse p65 (sense 5′- TGGGAAACCGTATGAGCCTG -3′ and antisense 5′- CCCGGAGTTCACTCATAGTTGT -3′), and mouse CD18 (sense 5′- GGGACTTGTCTTCCGACCTG -3′ and antisense 5′- GTGGAACCTCTTGGGACTCG -3′).

### Isolation of neutrophils from spleen

Neutrophils were isolated from the spleens of 10-week-old B6 mice using a neutrophil isolation kit (Miltenyi Biotec, Germany). To evaluate CD18 gene expression levels, the isolated neutrophils were cultured in RPMI-1640 medium either alone or supplemented with conditioned medium in a 1:1 ratio, prepared from the supernatant of MB49 cells 24 h after 5 Gy irradiation.

### FACS analysis

MB49, MBT2, LLC, H22, and Hepa 1–6 ectopic tumors were harvested and enzymatically digested using a cocktail consisting of collagenase (Gibco), DNase I (Thermo Scientific), and Dispase II (Gibco). The resulting single-cell suspensions were filtered through a 70 μm cell strainer (Falcon) and incubated in flow cytometry staining buffer (Invitrogen). The cells were then washed in FACS buffer (Ca^2+^-free PBS containing 3% FBS), followed by staining with the following antibodies: FITC-conjugated anti-CD45, BB700-conjugated anti-CD11b, BUV 395-conjugated anti-Ly6G, PE-conjugated anti-Ly6C, PAC-conjugated anti-CXCR2, PE-conjugated anti-CD18, PE-conjugated anti-ICAM1, and Alexa Fluor 647-A-conjugated anti-CD206 (BD Biosciences, La Jolla, CA, USA). After washing, the samples were analyzed with a BD FACSCalibur Cell Analyzer (BD Biosciences, La Jolla, CA, USA) and FlowJo V10.8.1 software. Flow cytometry analyses were performed with at least three independent biological replicates per group, and quantitative comparisons between groups were conducted using the Mann–Whitney *U* test.

### Cytokine array analysis

MB49 and MBT2 cells were cultured for 24 h with or without exposure to 5 Gy irradiation. The culture supernatants were collected and centrifuged at 1200 rpm for 5 min at 4 °C. Cytokine levels were analyzed via a Proteome Profiler™ Mouse XL Cytokine Array (R&D Systems, Minneapolis, MN, USA) according to the manufacturer’s instructions. The array membrane was visualized using MultiGel-21 (EBL Biotechnology, Taipei, Taiwan). The signal produced was proportional to the amount of analyte bound.

### Scratch wound healing assay

Cell migration was evaluated using a scratch wound healing assay. Confluent monolayers were scratched with a sterile pipette tip to create a uniform gap, washed with PBS to remove debris, and cultured in serum-reduced medium. Phase-contrast images were captured at baseline (0 h) and at indicated time points. Wound closure was quantified in ImageJ by measuring the wound width (W) at multiple positions across the gap. The percentage of closure was calculated as Closure% = (W₀ − Wₜ) / W₀ × 100, where W₀ is the initial wound width at 0 h and Wₜ is the wound width at time t. At least three independent wounds were analyzed per condition, and mean values were reported.

### Cell migration assay

Cell migration was assessed using 24-well Boyden chambers (8 μm pore size; Corning Incorporated, Corning, NY, USA). A total of 1 × 10^5 cells in serum-free RPMI were seeded into the upper chamber without Matrigel coating, and 500 μL RPMI was added to the lower chamber. After 4 h incubation at 37 °C, chambers were irradiated with 5 Gy or left untreated. At 48 h post-irradiation, non-migrated cells were removed, and migrated cells on the lower surface were fixed and stained with 0.5% crystal violet. Cells were counted in five random 200 × microscopic fields, and the average was used for analysis.

### RNA-seq data processing and bioinformatics analysis

Purified RNA was used to prepare a sequencing library via the TruSeq Stranded mRNA Library Prep Kit (Illumina, San Diego, CA, USA) following the manufacturer’s protocol. Briefly, mRNA was purified from total RNA (1 µg) using oligo (dT)-coupled magnetic beads and fragmented at elevated temperature. First-strand cDNA was synthesized using reverse transcriptase and random primers. Following the synthesis of double-strand cDNA and adenylation of the 3’ ends of the DNA fragments, adaptors were ligated. The resulting products were amplified via PCR and purified with the AMPure XP system (Beckman Coulter, Beverly, USA). Library quality was assessed using the Qsep400 System (Bioptic Inc., Taiwan) and quantified with a Qubit 2.0 fluorometer (Thermo Scientific, Waltham, MA, USA). The qualified libraries were sequenced on an Illumina NovaSeq 6000 platform, and 150 bp paired-end reads were produced by Genomics, BioSci & Tech Co., New Taipei City, Taiwan. Low-quality bases and adapter sequences in the raw data were removed using the fastp program (version 0.20.0) [13]. The filtered reads were aligned to the reference genome using HISAT2 (version 2.1.0) [14]. Gene abundance was quantified with FeatureCounts (v2.0.1) from the Subread package [15]. Differentially expressed genes (DEGs) were identified via either DESeq2 (version 1.28.0) [16] or EdgeR (version 3.36.0) [17], depending on the availability of biological replicates. Functional enrichment analysis of Gene Ontology (GO) terms among gene clusters was performed using the R package clusterProfiler (version 4.0.0) [18–20], and the results were visualized with SRplot [21].

### NanoString assay of patient samples

Total tumor RNA was extracted from three to six formalin-fixed, paraffin-embedded tumor samples, each of which was sectioned at a thickness of 10 μm. A total of 200–500 ng of RNA was extracted, with more than 20% of the RNA fragments being longer than 300 nucleotides (DV300 > 20%). The RNA samples were analyzed using the NanoString PanCancer Immuno-Oncology Panel (IO360), which includes 770 genes involved in the interaction between tumors, the microenvironment and the immune response in cancer. After hybridization with the Immune Profiling Panel by the NanoString Prep Station (NanoString Technologies, Seattle, WA, USA), sample analysis was performed on an nCounter Digital Analyzer (NanoString Technologies, Seattle, WA, USA). Raw data processing, quality control, and normalization were conducted using the nSolver 4.0 analysis software (NanoString Technologies, Seattle, WA, USA), with normalization to 40 housekeeping genes. All procedures, including preparation, hybridization, detection, scanning, and normalization, were performed according to the manufacturer’s instructions by Cold Spring Biotech, Taiwan.

## Results

### Local RT to neutrophil-rich metastatic tumors triggers new metastases in patients with stabilized metastatic urothelial carcinoma under systemic therapy

**Fig. 1:**
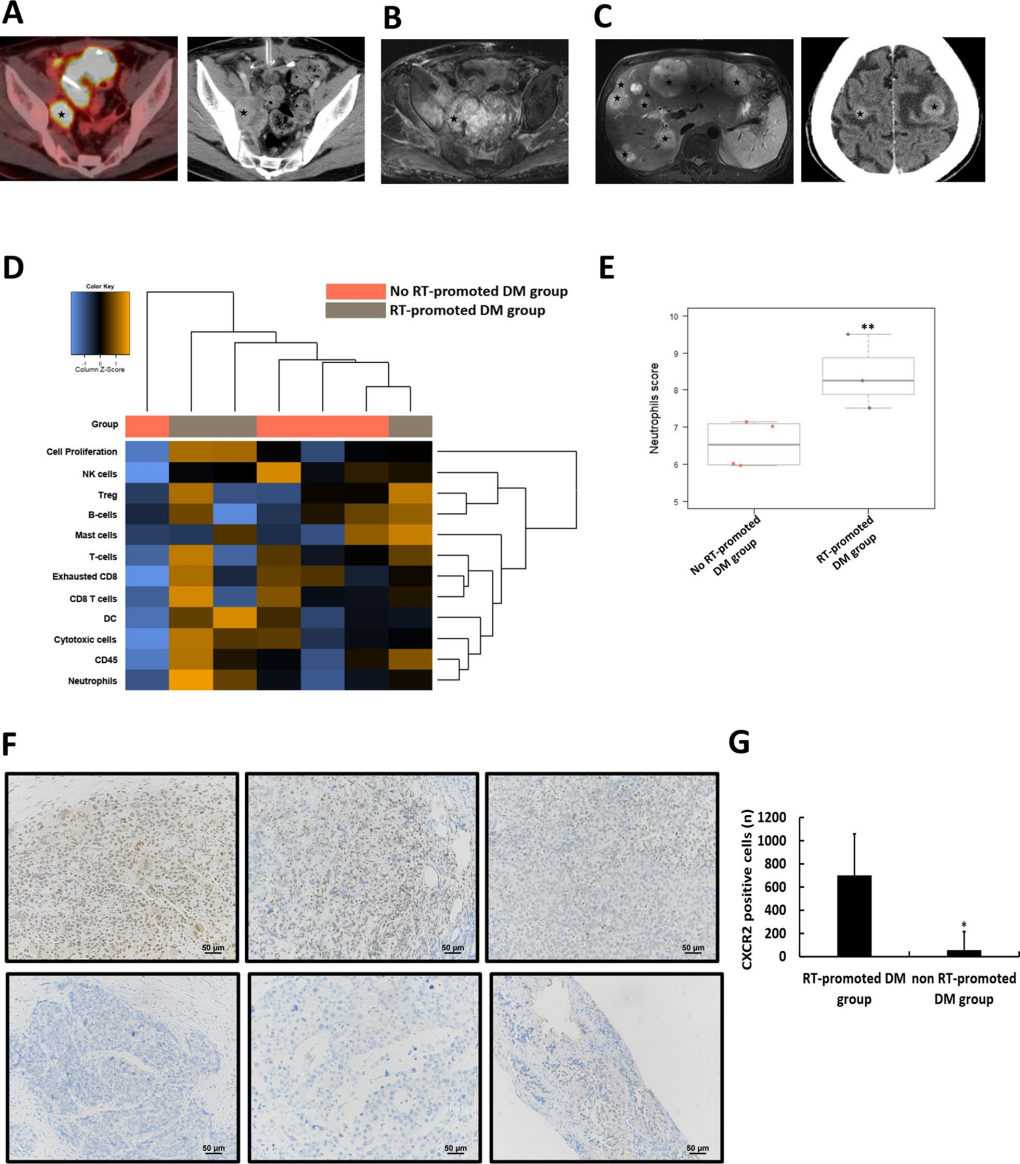
Intratumoral neutrophil infiltration is associated with patients who develop radiation (RT)-promoted distant metastasis (DM). Patient no. 1 in the RT-promoted DM group had residual pelvic lymphadenopathy (LAP), indicated by an asterisk, as seen on both the positron emission tomography (PET) scan (**A**, left) and the computed tomography (CT) scan (**A**, right). Two months after RT, the irradiated LAP remained stable (**B**); however, CT scans revealed new liver metastases (**C**, left, asterisk) and brain metastases (**C**, right, asterisk). **D** Heatmap of immune gene expression profiling via the nCounter Analysis System. **E** Neutrophil scores generated using advanced analysis via nSolver software. **F** Representative microscopy images (200 ×) of immunohistochemically stained urothelial tumor tissue sections of patients with RT-promoted DM (upper row) or no RT-promoted DM with CXCR2 are displayed (lower row), accompanied by (**G**) the corresponding cell counts. The values are presented as means ± SDs. *P < 0.05; **P < 0.01

To investigate the potential intrinsic factors related to either tumor cell or immune cell characteristics within the TME that could predict RT-promoted DM, we analyzed the transcriptomic differences in pretreatment tissues from 7 patients with urothelial cancer via NanoString analysis. All the patients received systemic treatment and had residual stabilized metastatic tumors treated with RT, primarily to the metastatic lymphadenopathies (Table S1, Fig. 1A, Fig. S1A and Fig. S1D). However, three of them, despite having smaller or stabilized irradiated lesions (Fig. 1B, Fig. S1B and Fig. S1E), developed new DMs in addition to their pre-RT stabilized metastatic lesions (Table S1, Fig. 1C, Fig. S1C and Fig. S1F); these three patients composed the RT-promoted DM group, whereas the other four composed the non-RT-promoted DM group. Cell type analysis (Fig. 1D, Fig. S2A-S2K) revealed that the neutrophil score was significantly higher for the RT-promoted group than the non-RT-promoted group (Fig. 1E). Given the role of C-X-C motif chemokine receptor 2 (CXCR2), a well-established surface marker of neutrophils [22, 23], we expanded our analysis to 30 patients with urothelial cancer (6 patients with and 24 patients without RT-promoted DM), and conducted IHC staining for CXCR2. Increased immunoreactivity of CXCR2 was associated with a significantly increased risk of RT-promoted DM (p = 0.005). The representative figures show more CXCR2-expressing cells in patients in the RT-promoted DM group (Fig. 1F, upper row) than the non-RT-promoted DM group (Fig. 1F, lower row), with quantification supporting those findings (Fig. 1G). Furthermore, survival analysis revealed that patients with CXCR2-positive tumors (≥ 5% of tumor cells) had significantly shorter overall survival compared with those with CXCR2-negative tumors (Fig. S1G). However, there were no significant differences in the pre-RT neutrophil-to-lymphocyte ratio (NLR) (Fig. S1H), pre-RT neutrophil percentage (Fig. S1I), or pre/post-RT percent changes in neutrophils from the peripheral blood between the two groups (Fig. S1J). Our findings from NanoString and IHC analyses suggest that intrinsic intratumoral neutrophil infiltration, but not the percentage of neutrophils in peripheral blood, is associated with an increased risk of RT-promoted DM. These clinical findings prompted us to validate the role of neutrophil infiltration as a predisposing factor in experimental mouse models.

### Neutrophil-rich ectopic tumors are associated with the increased risk of RT-promoted DM in different mouse models

**Fig. 2:**
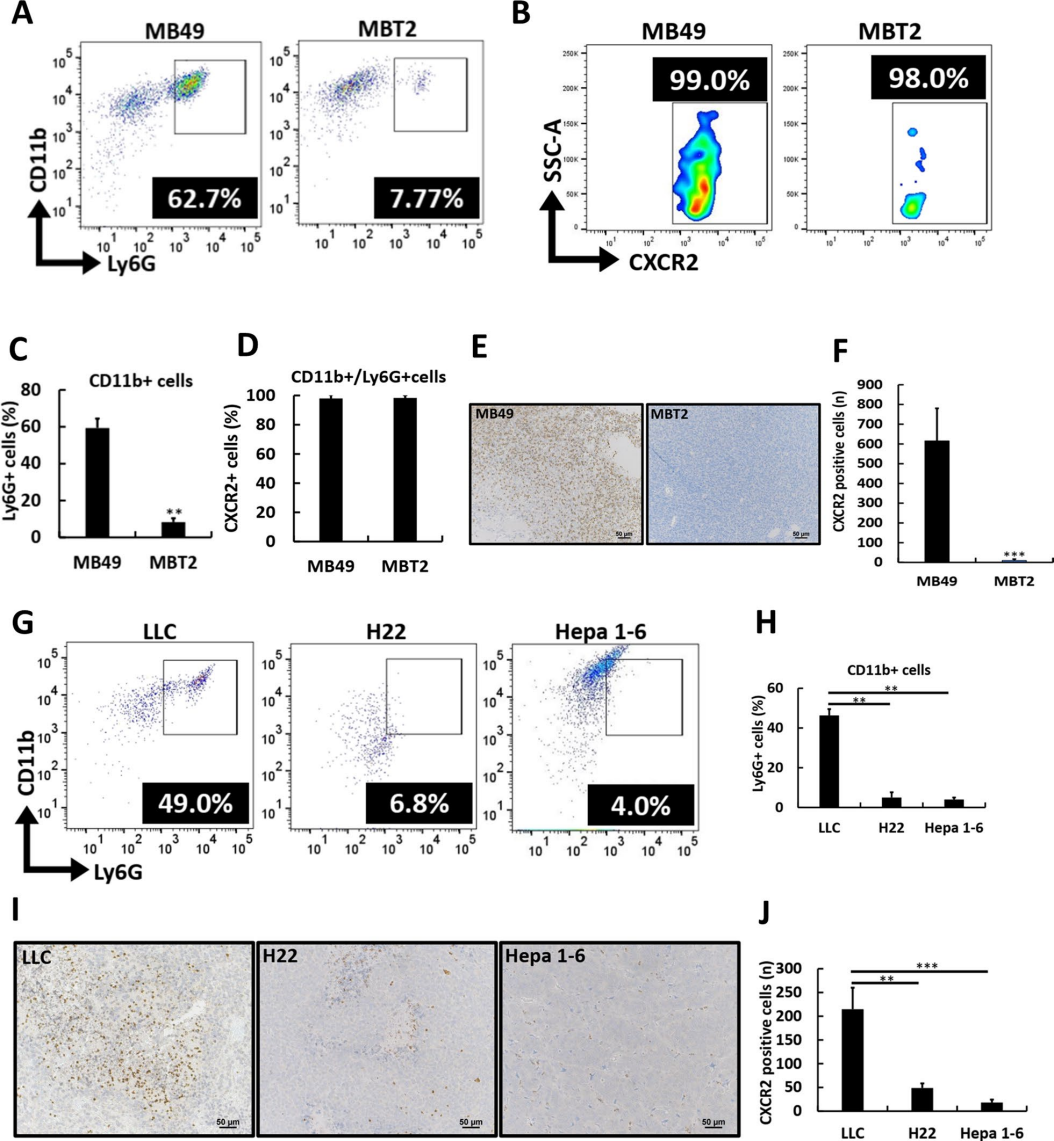
Increased numbers of CXCR2-expressing intratumoral neutrophils are associated with radiation (RT)-promoted distant metastasis (DM) in murine models. **A** Flow cytometry analyses of neutrophils, defined as CD11b + /Ly6G + cells, in MB49 (left) and MBT2 (right) mouse models are shown in representative images, along with the respective percentages of positive cells (**C**). A representative flow cytometry analysis (**B**) displays the percentages of CXCR2-positive neutrophils (**D**) in ectopic MB49 and MBT2 tumors. Representative microscopy images (200 ×) of immunohistochemically stained ectopic tumor tissue sections from MB49 (left) and MBT2 mouse models are shown (**E**), along with the corresponding cell counts (**F**). Representative flow cytometry images depicting the neutrophil populations (**G**) and their percentages (**H**) in the different ectopic tumors of LLC, H22, and Hepa 1–6 mouse models are shown. Microscopy images (200 ×) of immunohistochemically stained ectopic tumor tissue sections from LLC, H22, and Hepa 1–6 models are displayed (**I**), accompanied by the corresponding cell counts (**J**). The values are presented as means ± SDs. *P < 0.05; **P < 0.01; ***P < 0.001

**Fig. 3:**
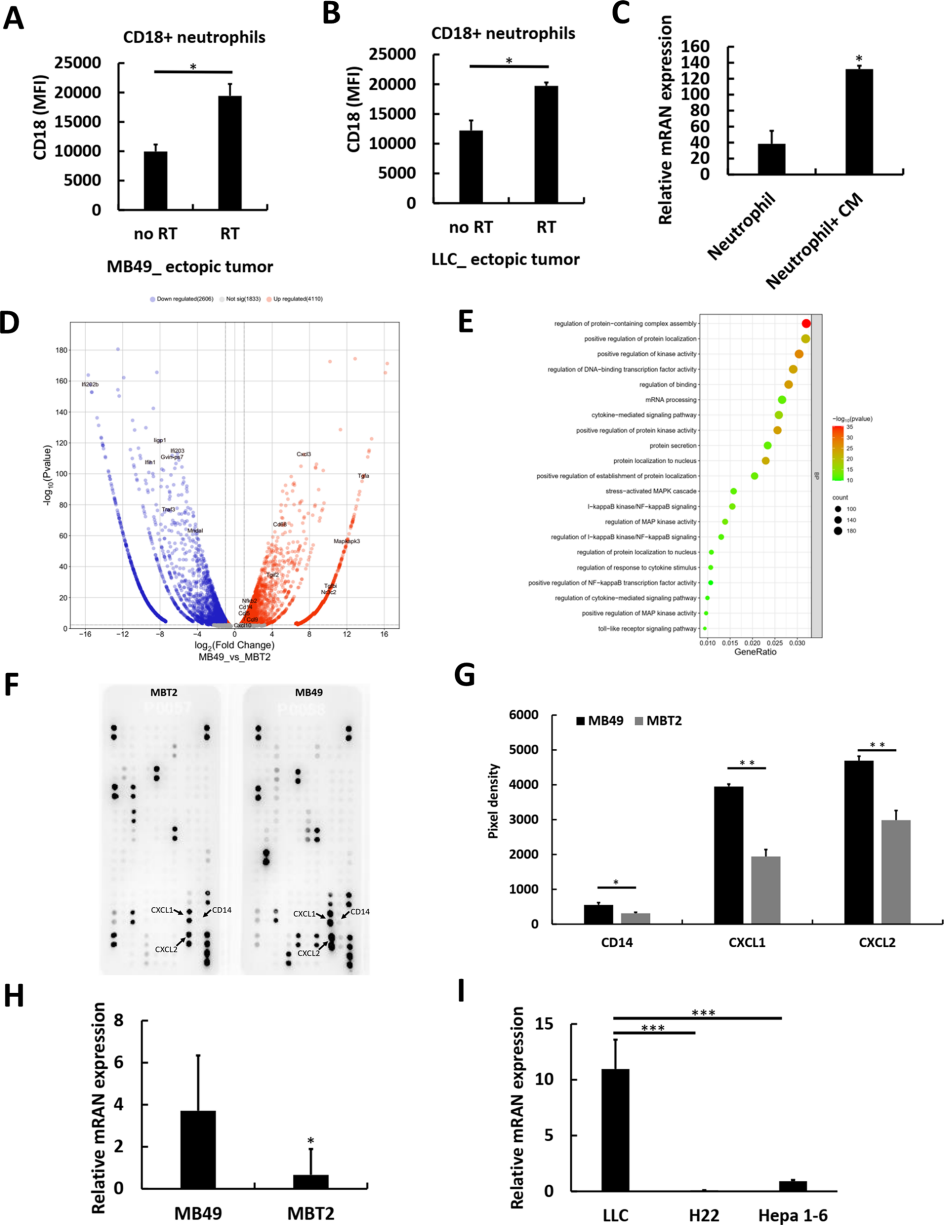
High CD14 expression in cancer cells drives neutrophil recruitment via cytokine production. CD18 expression intensities in intrinsic ectopic tumors and ectopic tumors 7 days after irradiation (7.5 Gy for 3 daily fractions) in both (**A**) MB49 and (**B**) LLC mouse models. **C** Real-time quantitative reverse transcription (qRT–PCR) analysis of CD18 expression on neutrophils isolated from the spleens of B6 mice, with or without conditioned medium (CM), derived from the supernatant of MB49 cells at 24 h after 5-Gy irradiation. **D** Volcano plot illustrating both upregulated and downregulated differentially expressed genes in the comparison between MB49 and MBT2 cells. **E** Dot plot showing Gene Ontology (GO) enrichment analysis of upregulated genes in the MB49 versus MBT2 cell lines. **F** Cytokine array analysis of culture medium from the MB49 (right) and MBT2 (left) cell lines, along with the expression levels of neutrophil-recruiting chemokines in the two cell lines (**G**). qRT–PCR analysis of CD14 expression in (**H**) MB49 and MBT2 cells as well as in (**I**) LLC, H22, and Hepa 1–6 cells. The values are presented as means ± SDs. *P < 0.05; **P < 0.01; ***P < 0.001. MFI: median fluorescence intensity

To investigate whether neutrophil infiltration is a key predisposing factor for an increased risk of RT-promoted DM in mouse models, we compared two established bladder cancer mouse models: the MB49-B6 model, which has a high risk of LM after RT to local ectopic tumors [5, 6], and the MBT2-C3H model [12], which follows a similar RT protocol (Fig. S3A) and exhibits local tumor control (Fig. S3B) but no LM after RT to ectopic tumors (Fig. S3C). Additional wound healing (Fig. S3D, S3E) and migration assays (Fig. S3F) showed no significant differences in the invasiveness capability of MB49 and MBT2 cells after RT. Flow cytometry analysis revealed significantly greater neutrophil levels, identified as CD11b + /Ly6G + cells (Fig. S3G), in MB49-B6 ectopic tumors than in MBT2-C3H ectopic tumors (Fig. 2A, C). CXCR2 expression was observed in over 95% of neutrophils across both models (Fig. 2B, D, S3G), with no detectable differences, supporting the use of CXCR2 as a reliable marker for neutrophil quantification in IHC analysis. In addition, IHC analysis revealed a significant increase in the number of CXCR2-positive cells in MB49-B6 ectopic tumors compared with MBT2-C3H ectopic tumors (Fig. 2E, F). To address concerns related to the difference in mouse species between the MB49 and MBT2 models, we employed an additional ectopic B6 mouse model using LLC cancer cells [6, 24], which displayed a similar pattern of RT-promoted LM to the MB49-B6 model. Besides, we used two other B6 mouse models with Hepa 1–6 and H22 cancer cells, and neither of which developed LM after RT. Similar to MB49-B6 ectopic tumors, LLC-B6 ectopic tumors presented increased neutrophil infiltration compared with both Hepa 1–6 and H22 ectopic tumors, as shown by flow cytometry (Fig. 2G, H). In addition, IHC analysis revealed higher CXCR2 expression in LLC-B6 ectopic tumors than Hepa 1–6 and H22 ectopic tumors (Fig. 2I, J). Regarding the potential pro-metastatic effects of neutrophils following RT, we observed a significant increase in CD18 expression intensity on neutrophils, a key integrin involved in neutrophil adhesion and cancer cell extravasation, in post-irradiated ectopic xenografts compared to non-irradiated xenografts of both neutrophil-rich MB49 and LLC tumors with post-RT LM (Fig. 3A, B, Fig. S4A-S4C). Additionally, in neutrophils extracted from the spleens of B6 mice, CD18 gene expression increased after exposure to conditioned medium (the supernatant from MB49 cells irradiated with 5 Gy) (Fig. 3C). CD18 on neutrophils facilitates tumor cell extravasation through binding with ICAM1 on tumor cells [25, 26], which was also shown with the higher expression with RT than without RT in these models (Fig. S4D, S4E). Furthermore, direct comparison of irradiated ectopic tumors and matched LM in the MB49 model revealed similar levels of neutrophil infiltration (Fig. S4F), with neutrophils in both sites consistently exhibiting high CXCR2 expression (Fig. S4G) and comparable CD18 intensity (Fig. S4H). This indicates that neutrophil accumulation and activation are preserved in metastatic lesions. Notably, time-dependent flow cytometry of MB49 ectopic tumors (control, post-RT day 7, post-RT day 14) showed no significant changes in N1 (ICAM1⁺), N2 (CD206⁺), or the N1/N2 ratio (Fig. S4I-S4K), suggesting that RT-promoted metastasis is not driven by specific neutrophil subsets. Collectively, these findings suggest that the intrinsic infiltration of CXCR2-positive neutrophils in ectopic tumors predisposes mouse models to a higher risk of RT-promoted DM, with enhanced neutrophil-mediated extravasation following RT contributing to this phenomenon. Based on this, we next explored tumor-intrinsic mechanisms that could explain why certain tumors attract and sustain neutrophil enrichment.

### CD14 expression in tumors is linked to the increased production of neutrophil-recruiting chemokines

On the basis of data from patients and mouse models, we propose that specific tumor characteristics contribute to enriched neutrophil infiltration in the TME. To explore the potential traits of tumor cells, we compared the transcriptomes of untreated MB49 and MBT2 cancer cells via RNA sequencing (RNA-seq). Compared with those in MBT2 cells, nuclear factor kappa B (NFκB) signaling pathway activation and the expression of its downstream products, such as C-X-C motif chemokine ligand 3 (CXCL3), were higher in MB49 cells (Fig. 3D, E, Fig.S5A). Notably, the expression of CD14, a toll-like receptor (TLR)-4-associated protein [27], was significantly upregulated in MB49 cells (Fig. 3D, Fig.S5A). CD14 plays a role in activating the TLR-4 signaling pathway, which in turn triggers NFκB signaling [27, 28]. Cytokine array analysis of the culture medium from unirradiated MB49 and MBT2 cells (Fig. S5B, S5C) revealed significantly increased concentrations of the CD14- and NFκB-associated neutrophil-recruiting chemokines CXCL1 and CXCL2 in MB49 cells (Fig. 3F, G). However, post-irradiation cytokine array showed no major differences between the two cell lines (Fig. S5D, S5E), suggesting that intrinsic chemokine differences were not sustained after RT**.** Consistently, in the MB49 mouse model, longitudinal ELISA analysis of serum samples demonstrated that CD14 and CXCL1 levels did not significantly change across RT time points or between mice with versus without LM (Fig. S5F, S5G), indicating that RT acts as a trigger enabling such traits to interact with immune or irradiated tumor cells to promote distant metastasis. Moreover, qRT‒PCR showed reduced CD14 gene expression in LM (Fig. S5H), and flow cytometry similarly revealed lower CD14 intensity in CD45⁻ tumor cells from LM compared with irradiated ectopic tumors (Fig. S5I), suggesting that LM may arise from CD14⁺ subpopulations of the primary tumor but display overall lower CD14 levels due to smaller tumor burden. Further qRT‒PCR analysis confirmed the increased gene expression of CD14 in MB49 cells compared with MBT2 cells (Fig. 3H), as well as LLC cells compared with H22 and Hepa 1–6 cells (Fig. 3I). These findings suggest that CD14-expressing cancer cells promote neutrophil-recruiting chemokine production via NFκB activation, leading us to investigate whether this CD14–NFκB–chemokine axis observed in vitro is also recapitulated in vivo within the tumor microenvironment.

### CD14-expressing cancer cells shape a neutrophil-enriched TME via NFκB-driven chemokine production

**Fig. 4:**
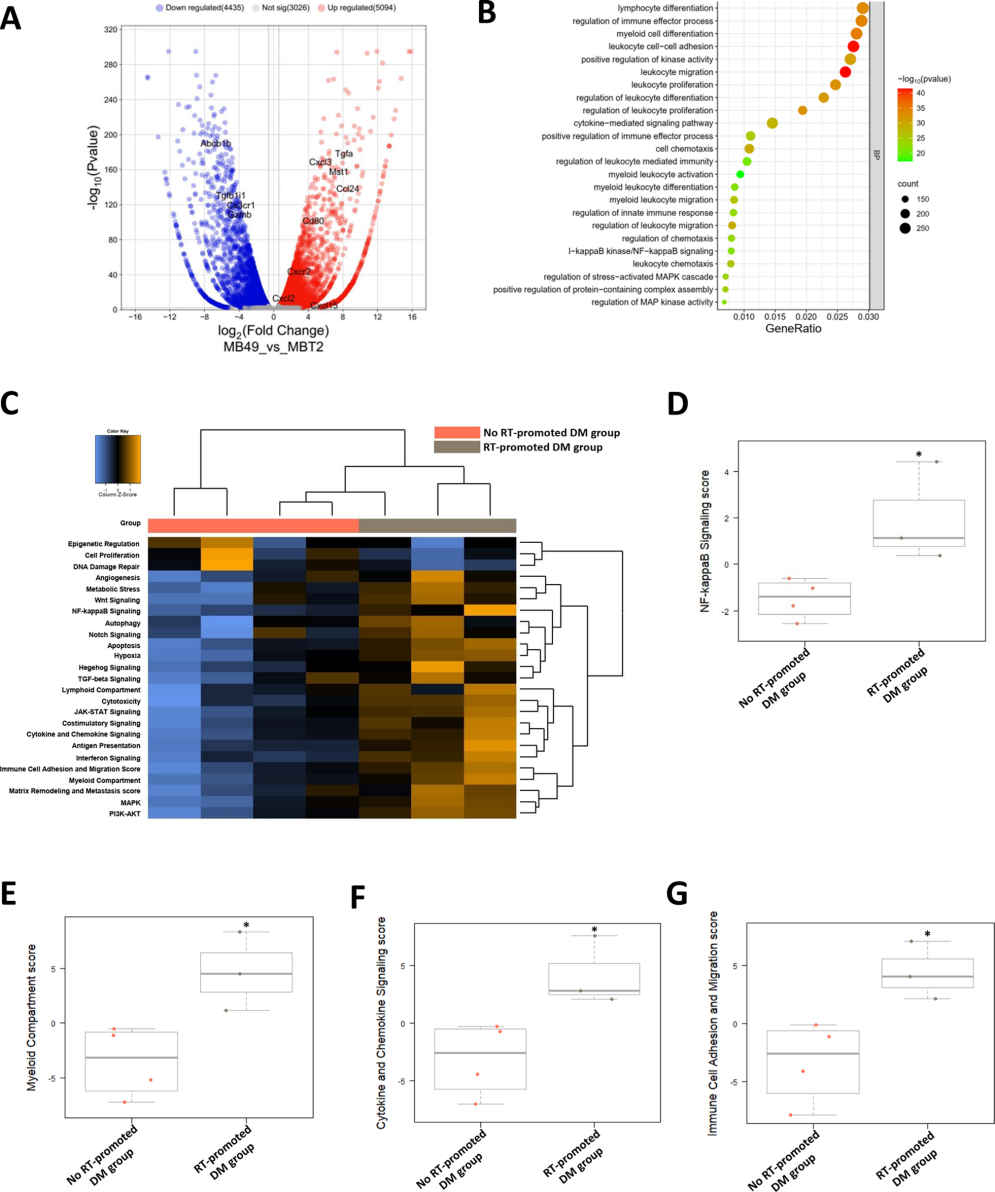
CD14-mediated nuclear factor kappa B signaling (NFκB) is associated with radiation-promoted distant metastasis in both murine models and patient groups. **A** Volcano plot illustrating both upregulated and downregulated differentially expressed genes in the comparison between MB49 and MBT2 ectopic tumors. **B** Dot plot showing Gene Ontology (GO) enrichment analysis of upregulated genes in MB49 versus MBT2 ectopic tumors. **C** Heatmap of signaling pathway profiling via the nCounter Analysis System. **D-G** Scores of signaling pathways related to neutrophil-recruitment chemokine production generated using advanced analysis with nSolver software. *P < 0.05. RT *radiation*, DM *distant metastasis*

To assess whether the in vitro correlation between CD14-expressing tumor cells and NFκB-driven chemokine production translates into the in vivo TME, we conducted RNA-seq analysis comparing MB49-B6 and MBT2-C3H ectopic tumors. This in vivo analysis confirmed the enhanced NFκB signaling pathway activation observed in vitro, indicating greater NFκB activation in MB49 cancer cells and their TME (Fig. 4A, B). Notably, we also detected increased activation of the neutrophil surface marker CXCR2 and neutrophil-related signaling pathways in MB49 tumors compared with MBT2 ectopic tumors (Fig. 4A, B). Consistent with the findings in the mouse model, NanoString analysis (Fig. 4C–G, Fig. S5J) revealed increased NFκB activation and neutrophil-associated chemokine signaling in patients in the RT-promoted DM group. The findings for mouse models and patients support the concept that high-CD14-expressing tumors drive NFκB activation-related chemokine production, leading to a neutrophil-enriched TME. Given the importance of neutrophil accumulation, we next tested whether targeting neutrophils themselves could mitigate badscopal effect.

### Blocking neutrophils with Ly6G or CXCR2 antagonists reduces the risk of RT-promoted DM in vivo

**Fig. 5:**
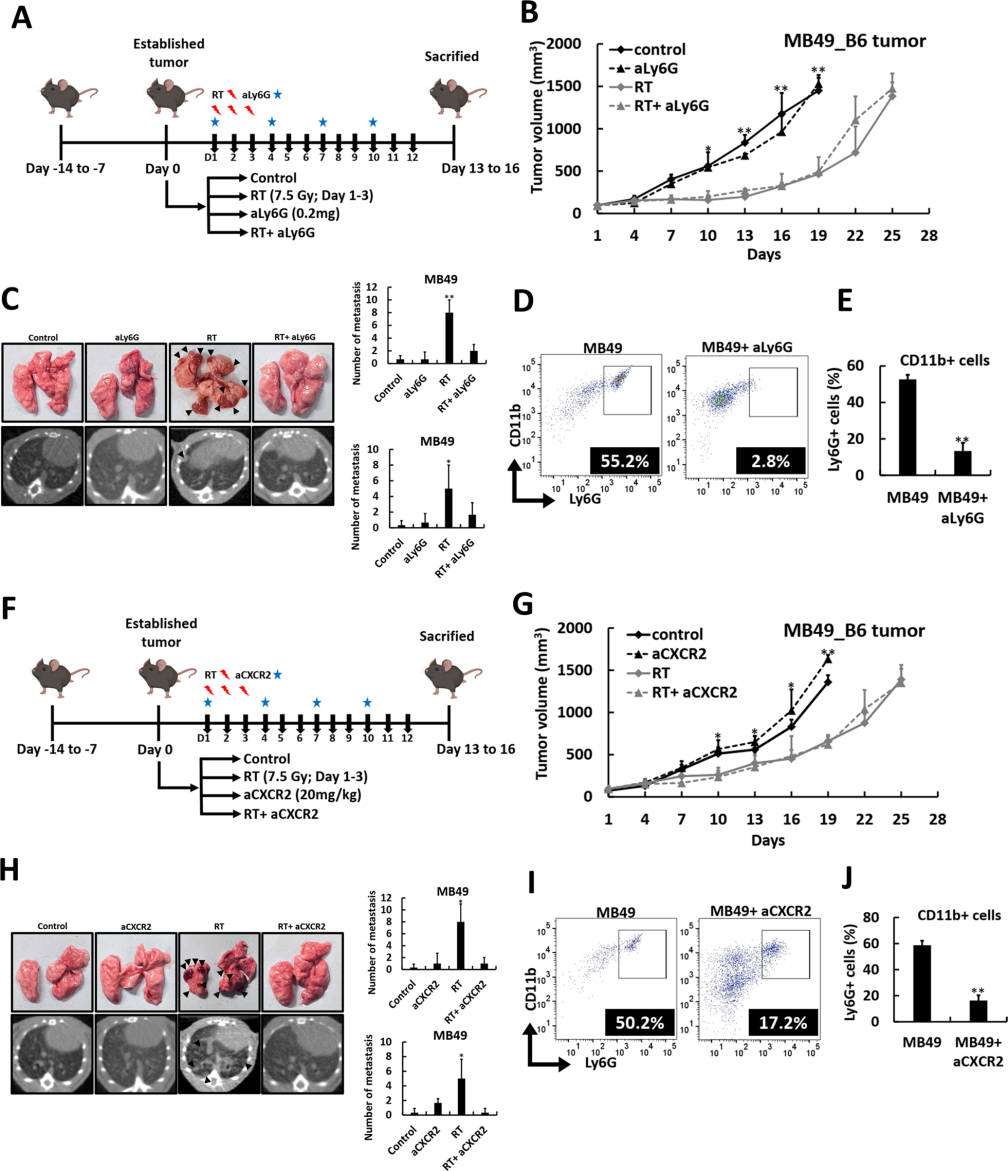
Blocking intratumoral neutrophil infiltration with neutrophil-depleting agents significantly reduces the risk of radiation-promoted distant metastasis. **A** The experimental workflow of the MB49 ectopic mouse model is outlined, comparing the treatment responses among the control group, the radiotherapy (RT) group, the Ly6G antagonist (aLy6G) group, and the RT plus aLy6G group. Tumor growth curves (**B**) and the number of pulmonary metastases, as indicated by gross surface lesions (**C**, upper row; arrow) and on computed tomography (CT) (**C**, lower row; arrow), were compared. A representative flow cytometry figure demonstrating the CD11b + /Ly6G + population is presented (**D**), along with the corresponding percentages (**E**) in MB49 ectopic tumors from the control and aLy6G groups. **F** The experimental workflow was similar for the MB49 ectopic mouse model; the treatment responses of the control group, the RT group, the CXCR2 antagonist (aCXCR2) group, and the RT plus aCXCR2 group were compared. Tumor growth curves (**G**) and the number of pulmonary metastases, as indicated by gross surface lesions (**H**, upper row; arrow) and on CT (**H**, lower row; arrow), were compared. A representative flow cytometry plot showing the CD11b + /Ly6G + population is presented (**I**), along with the corresponding percentages (**J**) of MB49 ectopic tumors among the control and aCXCR2 groups. The values are presented as means ± SDs. *P < 0.05; **P < 0.01. *RT* radiotherapy, *aLy6G* Ly6G antagonist, *aCXCR2* CXCR2 antagonist

To further investigate the role of neutrophil enrichment in badscopal effect, a Ly6G antagonist (aLy6G) was used in both MB49-B6 (Fig. 5A) and LLC-B6 mouse models (Fig. S6A). Neutrophil blockade did not impact local tumor control in either model (Fig. 5B, Fig. S6B) but significantly reduced the incidence of pulmonary metastasis (Fig. 5C, Fig. S6C). In addition, aLy6G treatment markedly decreased the intrinsic neutrophil infiltration of ectopic tumors in both models (Fig. 5D, E, Fig. S6D-S6E). Similarly, using a CXCR2 antagonist (aCXCR2) under the same experimental design (Fig. 5F, Fig. S6F), we observed a significant reduction in neutrophil infiltration (Fig. 5I, J, Fig. S6I-S6J), but the risk of RT-promoted DM was mitigated (Fig. 5H, Fig. S5H) without compromising local tumor control (Fig. 5G, Fig. S6G). chemokine networks inhibition failed to reduce neutrophil infiltration or prevent badscopal effect (Fig. S7A-S7D), underscoring that the redundant chemokine networks may limit the efficacy of single-chemokine blockade in CD14-high tumors. In addition, double IHC staining for CD11b and Ly6G in lung tissues showed that LM lesions were enriched in CD11b⁺ myeloid cells, while adjacent lung regions contained Ly6G⁺/CD11b⁺ double-positive neutrophils (Fig. S7E-S7G). Normal lungs without LM had very few such cells, suggesting neutrophils act together with other myeloid cells in the LM microenvironment. These results confirmed the pivotal role of neutrophil infiltration in contributing to RT-promoted DM. We next evaluated whether targeting CD14 in tumor cells could diminish neutrophil recruitment and thereby limit the badscopal effect.

### Inhibiting CD14 in tumor cells reduces RT-promoted LM by blocking neutrophil recruitment and infiltration

**Fig. 6:**
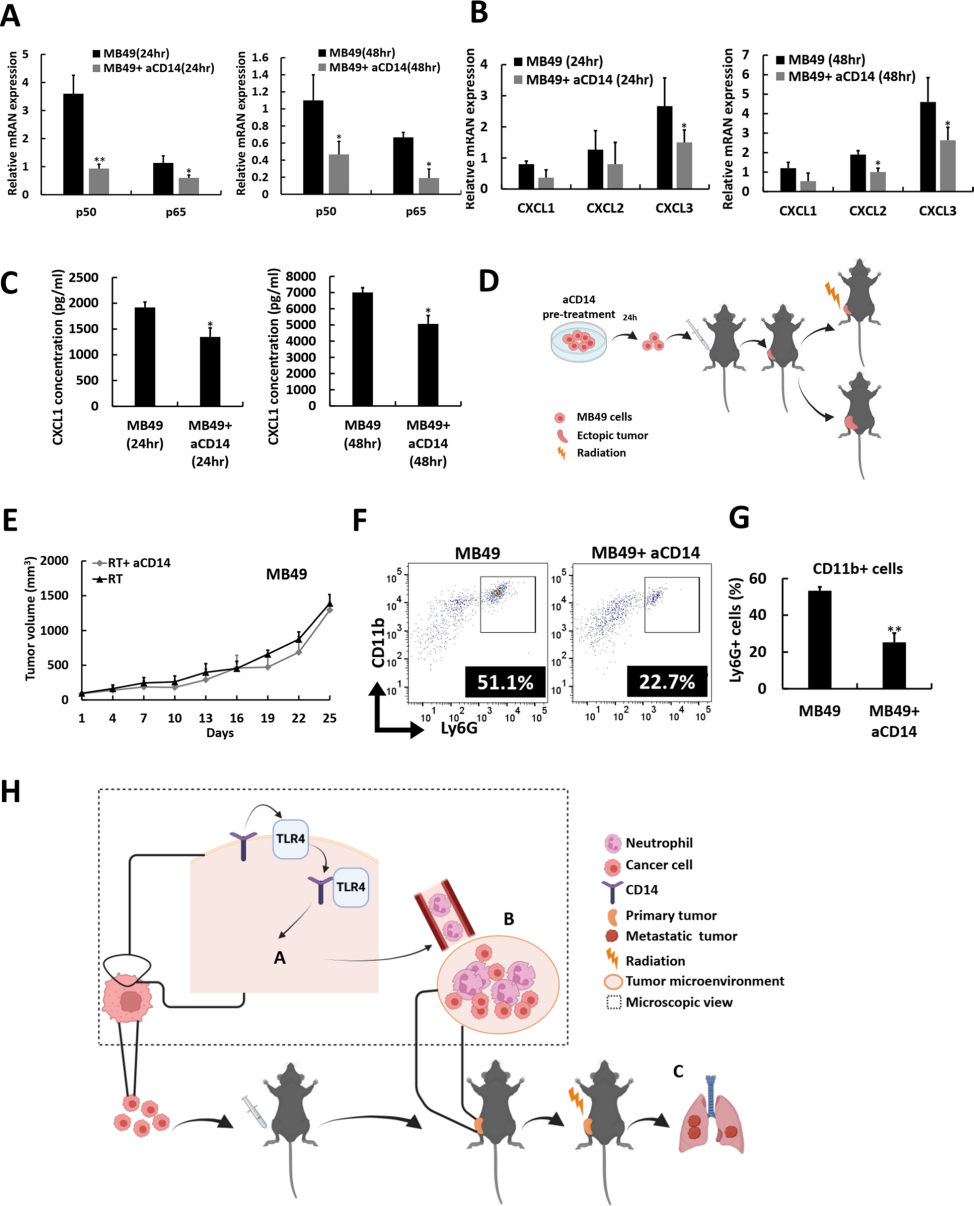
Inhibiting CD14 expression in tumor cells effectively counteracts radiation (RT)-promoted distant metastasis (DM) by diminishing nuclear factor kappa B (NFκB) activation, which leads to reduced neutrophil recruitment and intratumoral infiltration. Real-time quantitative reverse transcription (qRT–PCR) analysis of proteins associated with (**A**) NFκB signaling and (**B**) chemokines involved in neutrophil recruitment in MB49 cells pretreated with or without a CD14 antagonist (aCD14) for 24 h and 48 h. **C** Enzyme-linked immunosorbent assay of CXCL1, a neutrophil-recruiting chemokine, in MB49 cells pretreated with or without aCD14 for 24 h or 48 h. **D** In the experimental workflow, MB49 cells were pretreated with aCD14 for 24 h to investigate the effects of CD14 suppression on RT-promoted DM and neutrophil infiltration in ectopic tumors. **E** The tumor growth delay curves for the RT group generated with MB49 cells, with or without aCD14, are displayed, with day 0 referring to the day of tumor establishment (mean starting tumor volume = 90 mm^3^). **F** A representative flow cytometry analysis displays the percentage of neutrophils in ectopic MB49 tumors and aCD14-pretreated MB49 tumors, alongside (**G**) their quantified numbers. **H** A graphic summary of the main findings presented in the study (created using BioRender.com). **A**: CD14-high-expressing tumors drive the activation of NFκB signaling, which in turn initiates chemokine activation. **B**: The production of chemokines facilitates neutrophil recruitment and infiltration within the tumor microenvironment (TME). **C**: Such a neutrophil-infiltrated TME indicates a heightened risk of RT-promoted DM. The values are presented as means ± SDs. *P < 0.05; **P < 0.01. *RT* radiotherapy, *aCD14* CD14 antagonist

To explore the role of CD14 expression in creating a neutrophil-enriched TME, we employed a CD14 antagonist (aCD14) in both MB49 and LLC cells. After 24 and 48 h of aCD14 pretreatment, the gene expressions of the NFκB activation proteins p50 and p65, as well as the NFκB activation-related neutrophil-recruiting chemokines CXCL1, CXCL2, and CXCL3, were significantly reduced, as shown by qRT‒PCR analysis (Fig. 6A, B, Fig. S8A‒S8B). ELISAs also revealed decreased CXCL1 levels in the culture medium of both MB49 (Fig. 6C) and LLC cells (Fig. S8C). Furthermore, treatment with a TLR4 antagonist in MB49 cells similarly downregulated CD14, NFκB subunits (p50, p65), and related chemokines (CXCL1–3) (Fig. S8D), supporting that the CD14 effect is TLR4-dependent. In a mouse model using aCD14-pretreated MB49 cells (Fig. 6D, Fig. S8E), no RT-promoted LM was observed (Fig. S8F), and the local tumor control remained unaffected (Fig. 6E). Additionally, aCD14-pretreated MB49-B6 ectopic tumors (Fig. 6D) presented significantly decreased neutrophil infiltration (Fig. 6F, G). Similar results were obtained in the aCD14-pretreated LLC-B6 mouse model (Fig. S8G-S8L). These findings confirm that CD14 expression in cancer cells plays a critical role in shaping a neutrophil-enriched TME for the RT-promoted DM (Fig. 6H).

## Discussion

RT-promoted DM, known as the badscopal effect, is a rare but serious event that has not been observed in all types of tumors. However, several preclinical and clinical studies have reported cases of this phenomenon, suggesting that RT for certain cancer types might produce prometastatic and noninvasive signals [1–3, 29]. Several studies have demonstrated that sublethal RT to tumors can increase the invasive capability of surviving cancer cell descendants and trigger metastatic processes, including vasculogenesis, epithelial mesenchymal transition, and migration [29–33]. Concurrently, RT may also induce immunosuppressive phenotype in immune cells, such as lowering phagocytic function of myeloid populations [3]. When badscopal effect does occur, it can offset the effectiveness of RT in controlling local tumors and may worsen the prognosis of patients. Our previous findings demonstrated that post-RT CCL2 secretion by specific murine cancer cells, such as MB49 and LLC cells, contributes to the interactions with both tumor associated macrophages (TAMs) and Tregs in the post-irradiated TME, which increases the risk of badscopal effect [5, 6]. This study further demonstrated that a neutrophil-enriched TME is a critical factor in shaping a TME predisposed to RT-promoted DM, as observed in both patient samples and mouse models (Fig. 6J). Our findings offer valuable insights for clinical treatment strategies.

Neutrophils, the most abundant immune cells in circulation, play an essential role in the innate immune response, particularly in combating infections and inflammatory insults [34]. In addition, the NLR, a recognized marker of systemic inflammation, is a well-established index for predicting poor tumor prognosis [35] as well as the risk of lymph node and distant metastasis [36, 37]; however, the underlying mechanisms remain under investigation. Within the TME, neutrophils exhibit both protumor and antitumor properties, driven by their ability to express a wide repertoire of cytokines and engage in complex, bidirectional interactions with lymphoid cells and macrophages [7–9, 25, 38–40]. Moreover, neutrophils possess “plasticity”, a capacity to reprogram their transcriptome and epigenetics, allowing them to differentiate into discrete subsets and leading to their dual function within the TME [38, 39, 41]. Neutrophil infiltration into solid tumors varies across cancer types, and high levels of tumor-associated neutrophils are linked to poor prognosis in most, though not all, human tumors [34, 42]. Moreover, increasing evidence suggests that neutrophils play an active role in regulating the TME and promoting tumor metastasis [8, 9], notably through CD18-mediated extravasation [25, 26]. Our results align with these observations, demonstrating enhanced CD18 expression on neutrophils following RT. This increase facilitates the escort of tumor cells into bloodstream through directly binding ICAM1 on their surface, and ultimately leads to LM in both the inherently neutrophil-enriched MB49 and LLC mouse models.

Of note, the chemokine receptor CXCR2 on neutrophils plays a critical role in regulating neutrophil recruitment [25] by interacting with ligands from the C-X-C family, including CXCL1, CXCL2, and CXCL3 [22, 23, 43, 44]. These chemokines drive neutrophil chemotaxis through the CXCL-CXCR2 signaling axis [43], which in turn contributes to metastasis [45] and reduces the efficacy of immunotherapy [46]. The overexpression of CXCL1 and CXCL2 in cancer cells has been shown to attract neutrophils into tumors, leading to chemoresistance, a process that can be mitigated by blocking CXCR2 [45]. Additionally, CXCL1, CXCL2, and CXCL3 in tumors can be regulated by NFκB signaling [44, 45, 47], which has been identified as a crucial pathway for neutrophil recruitment [48, 49]. In this study, we found that intratumoral neutrophils are predominantly CXCR2 + , which is consistent with the role of CXCR2 in mobilizing neutrophils from the bone marrow and peripheral blood to the TME. Additionally, both patient specimens and ectopic tumors from different mouse models with neutrophil-enriched TMEs presented increased NFκB signaling activity and increased neutrophil-recruiting chemokine secretion. These findings suggest that NFκB activation is crucial in promoting neutrophil infiltration within the TME.

CD14, a differentiation antigen of monocytes, also functions as a coreceptor for TLR-4, together becoming activated by inflammatory signals such as lipopolysaccharides [27, 50]. The expression of CD14 facilitates TLR4 activation, which in turn triggers the NFκB signaling pathway [28]. While CD14 is typically expressed in immune cells, a high level of CD14 expression has been observed in bladder cancer cells and is associated with the increased infiltration of myeloid cells, including neutrophils, compared with other cancer cells with minimal CD14 expression [51]. In experiments comparing CD14-high and CD14-low MB49 cancer cells generated through serial FACS sorting and passaging, CD14-high cells exhibited greater enrichment of neutrophil-recruiting chemokines, such as CXCL1 and CXCL2, than did CD14-low cells [51]. CD14 expression has also been found to be upregulated in cancer stem cells to increase their proliferation, migration and metastatic capacity [52]. Our findings are in line with those results, demonstrating that CD14-high cancer cells promoted greater neutrophil accumulation in the TME than did CD14-low cancer cells. Moreover, inhibiting CD14 expression in tumor cells reduced the levels of neutrophil-recruiting chemokines by suppressing NFκB signaling, leading to a significant decrease in neutrophil infiltration within the TME.

One major limitation of this study is the uncommon RT-promoted DM, making it difficult to collect sufficient patient samples for confirmatory evaluation. To address this, we utilized RNA screening techniques, including NanoString analysis and RNA-seq, along with multiple mouse models exhibiting both the presence and absence of RT-promoted DM. However, when badscopal effects occur, patients often require timely systemic therapy and are not medically or ethically suitable candidates for biopsy of metastatic disease, which limits the availability of translational information from metastatic tumors for direct comparison. Therefore, prospective clinical trials are needed not only to further investigate and confirm the current preclinical findings through metastasis monitoring and serum biomarker assessment, but also to take the challenge of limited metastatic tumor sampling. Besides, our study focused on the intrinsic TME prior to RT by identifying patients at greater risk of RT-promoted DM, especially those with high CD14 expression and neutrophil-enriched tumors. However, we lack the defined thresholds for CD14 or neutrophil expression due to the limited availability of clinical samples, either from primary or metastatic tumors. Given that neutrophil blockade using a Ly6G antagonist successfully mitigated RT-promoted LM in our mouse models, this strategy is not feasible in clinical practice with the critical role of neutrophils in defending the body against infections. To address this limitation, we specifically targeted CXCR2-positive neutrophils, considering the link between CXCR2 expression and neutrophil migration from bone marrow to circulation [53, 54]. With the availability of CXCR2 antagonist Reparixin in clinical trials, we found that targeting CXCR2-positive neutrophils demonstrated the effect comparable to Ly6G antagonism, indicating the potential clinical feasibility of this strategy. Another limitation of this study is the absence of CD14-targeted genetic models. As a future goal, we plan to generate CD14-knockdown cell lines and tumor models to further validate its mechanistic role. However, because CD14 is also widely expressed on various immune cells, broad inhibition of CD14 would be impractical in the clinical setting. Therefore, unlike in our mouse models, direct genetic modification of CD14 expression in patient tumors may not be feasible. Hence, further investigation into how this neutrophil-enriched effect contributes to the increased risk of DM after RT is needed. Exploring factors such as changes in the neutrophil phenotype or interactions between neutrophils and TAMs after RT could lead to significant breakthroughs, and future studies will require multiplex IHC or spatial transcriptomics to dissect CD14 expression within tumor cells. Given the multifaceted role of neutrophils in the TME, it is crucial to further investigate their CD18–ICAM1-mediated extravasation as well as their effects on adaptive immunity and interactions with TAMs, particularly the roles of N1/N2 subpopulations.

## Conclusions

Our study highlights the pivotal role of CD14 expression in tumors in shaping a neutrophil-enriched TME through NFκB signaling. This neutrophil-enriched TME increases the susceptibility to RT-promoted DM. Inhibiting CD14 expression in tumor cells reduces neutrophil-recruiting chemokines, leads to decreased neutrophil infiltration in the TME, and consequently lowers the risk of RT-promoted DM.

## Supplementary Information

Supplementary material 1.

Supplementary material 2.

Supplementary material 3.

## Data Availability

The datasets used and analysed during the current study are available from the corresponding author on reasonable request.

## Abbreviations

RT: Radiation
CCL2: C–C motif chemokine receptor 2
CCR2: C–C motif chemokine receptor 2
CCR4: C–C motif chemokine receptor 4
Tregs: Regulatory T cells
TME: Tumor microenvironment
DM: Distant metastasis
LLC: Lewis lung carcinoma
B6: C57BL/6
C3H: C3H/H3N
IGRT: Image-guided radiotherapy
CBCT: Cone beam computed tomography
ELISA: Enzyme-linked immunosorbent assay
CXCR2: C-X-C motif chemokine receptor 2
CXCL: C-X-C motif chemokine ligand
TLR: Toll-like receptor
aLy6G: Ly6G antagonist
aCXCR2: CXCR2 antagonist
aCD14: CD14 antagonist
TAMs: Tumor associated macrophages
