# Supplementary figures and images for: High-CD14-expressing urothelial cancer cells foster a neutrophil-rich tumor microenvironment that increases the risk of radiation-promoted distant metastasis
Source: J Biomed Sci. 2026 Jan 4;33:2. doi: 10.1186/s12929-025-01201-2 (PMC12765301; doi:10.1186/s12929-025-01201-2)

# Figure S1

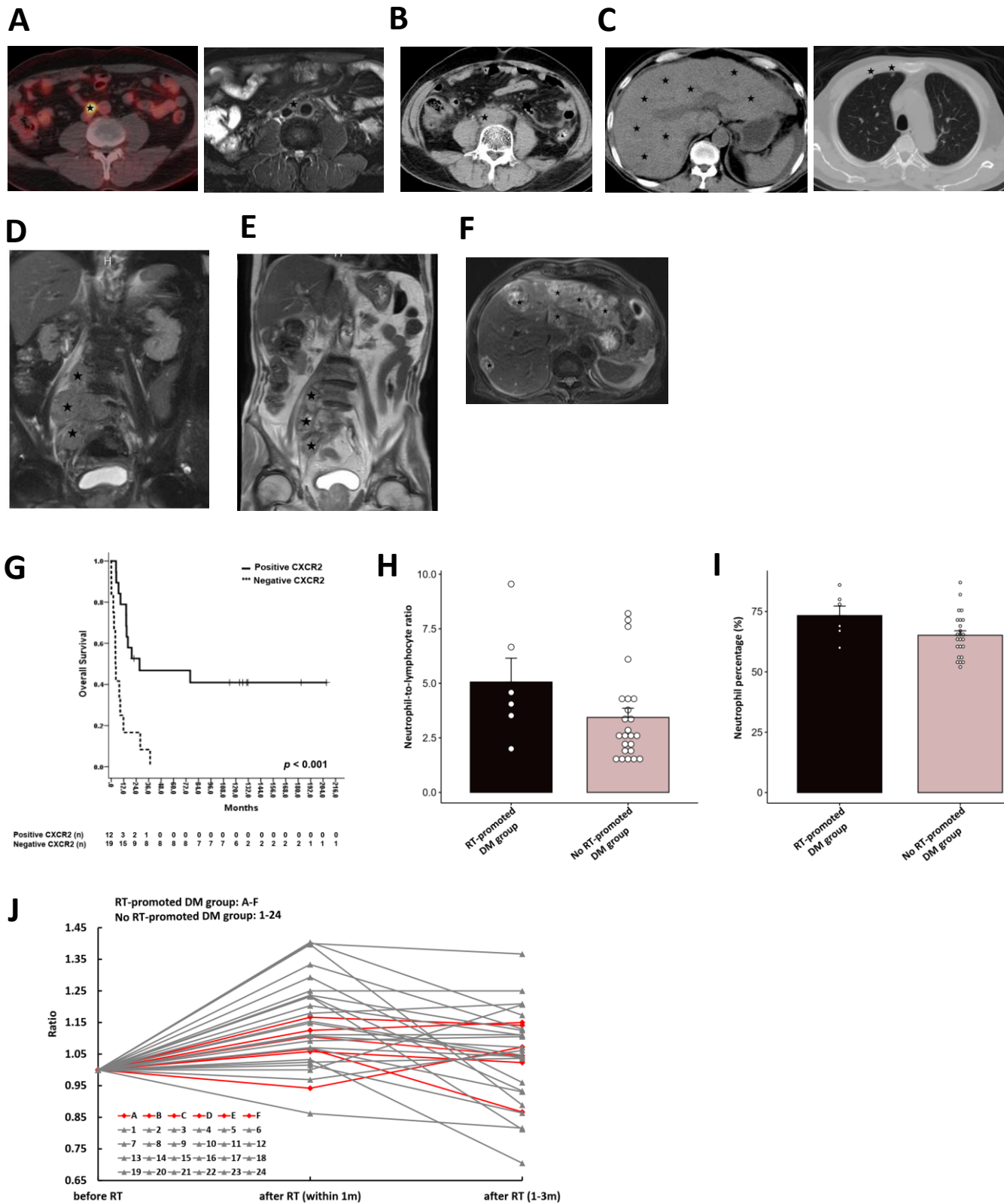

# Figure S2

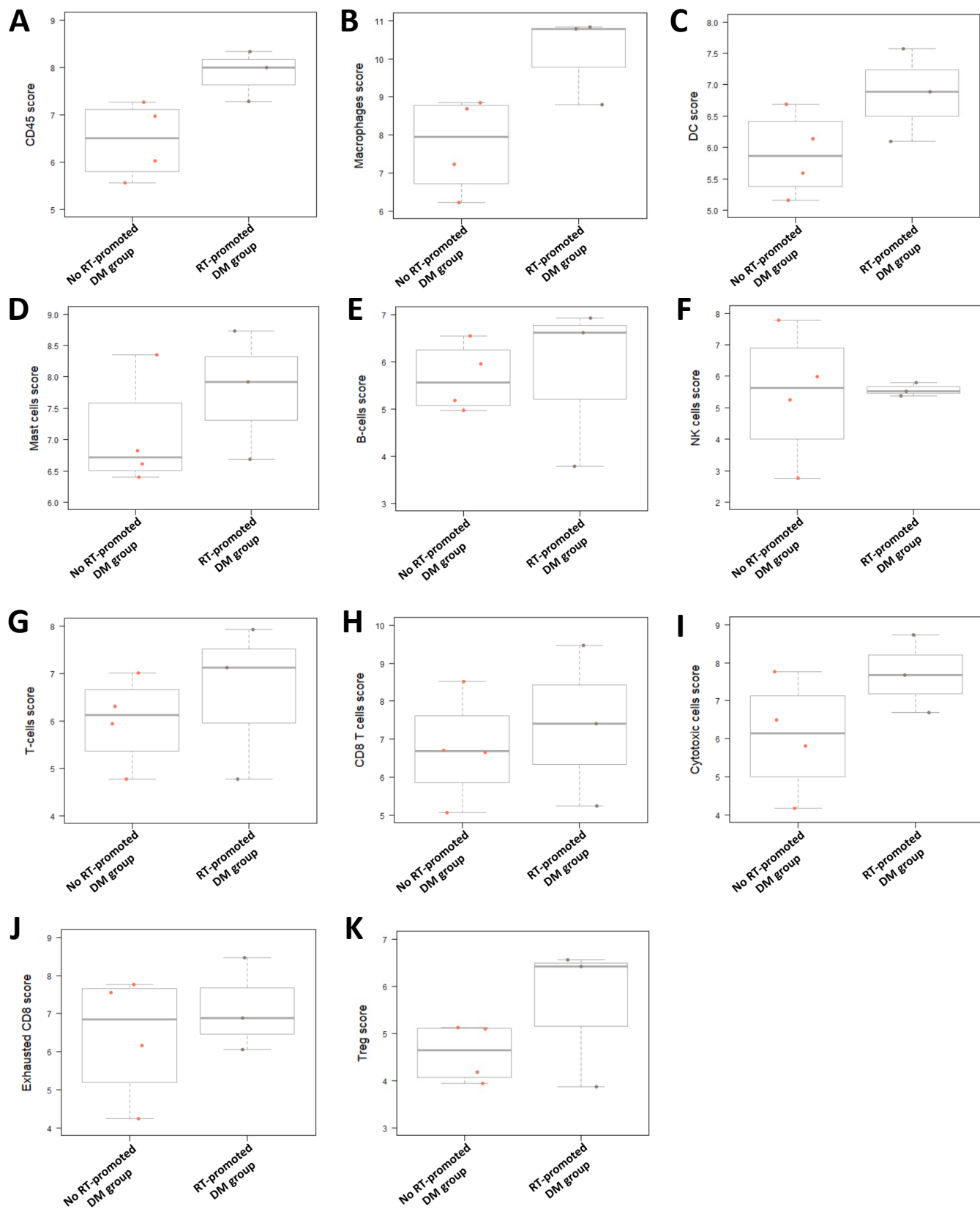

# Figure S3

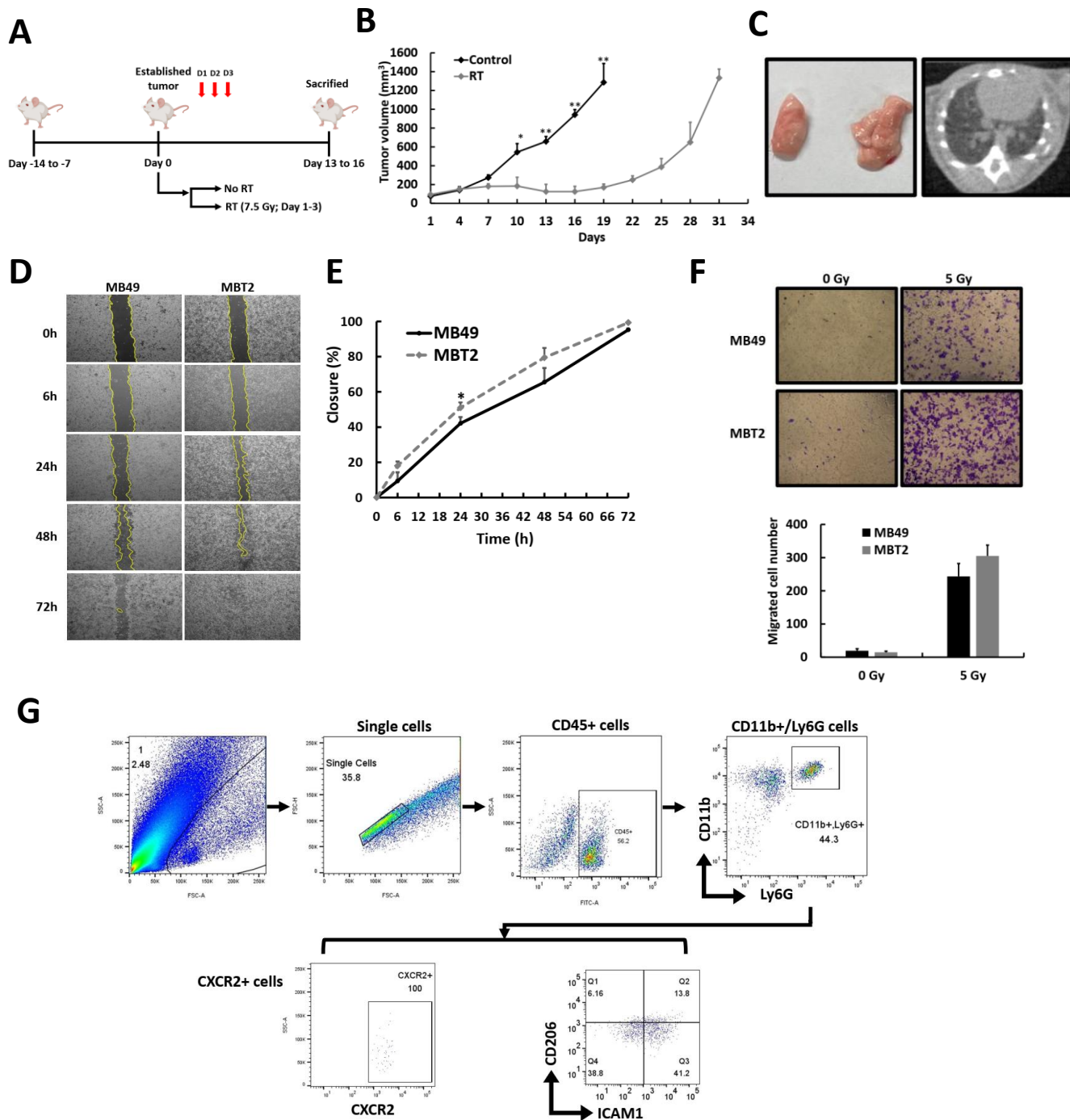

# Figure S4

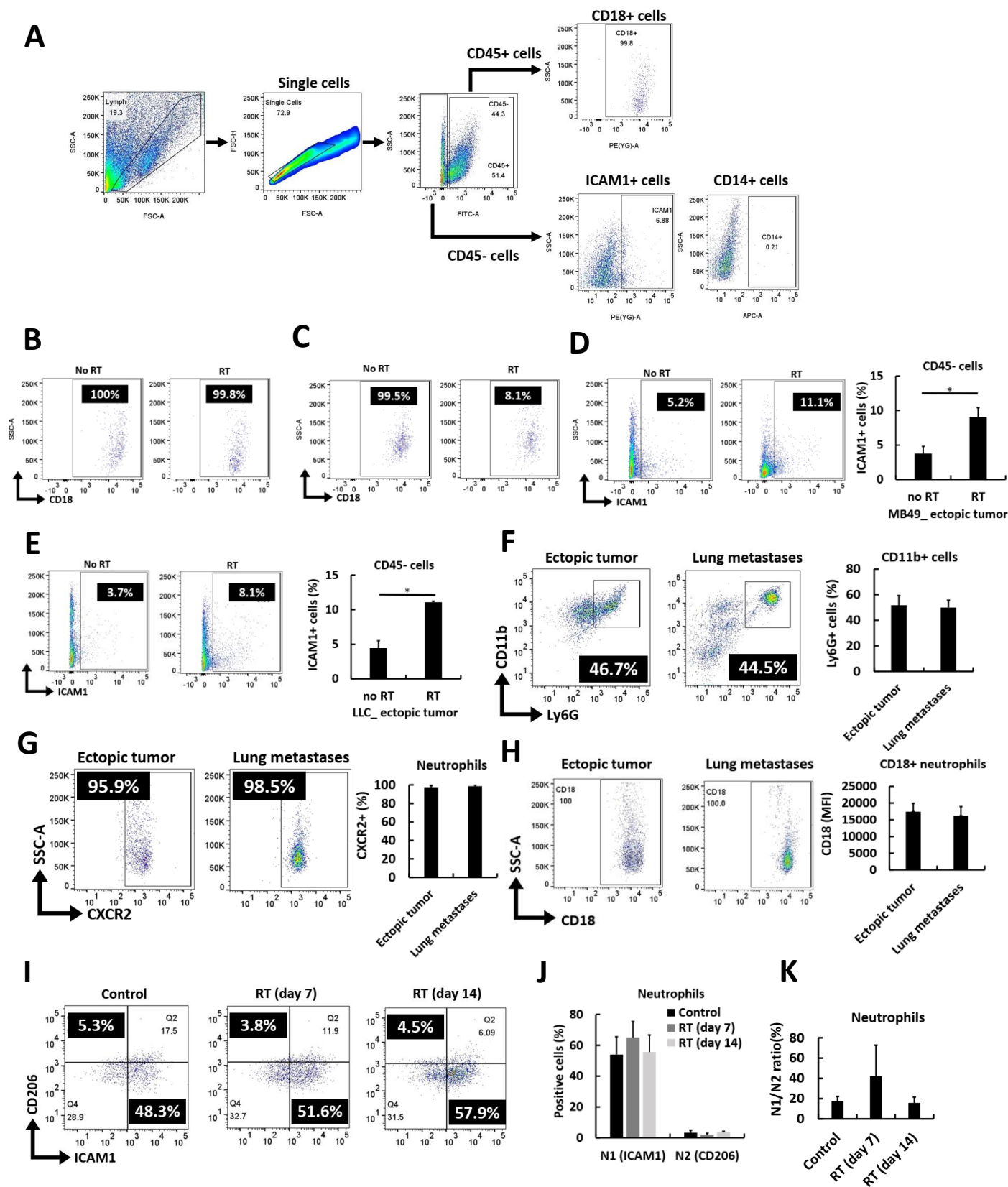

# Figure S5

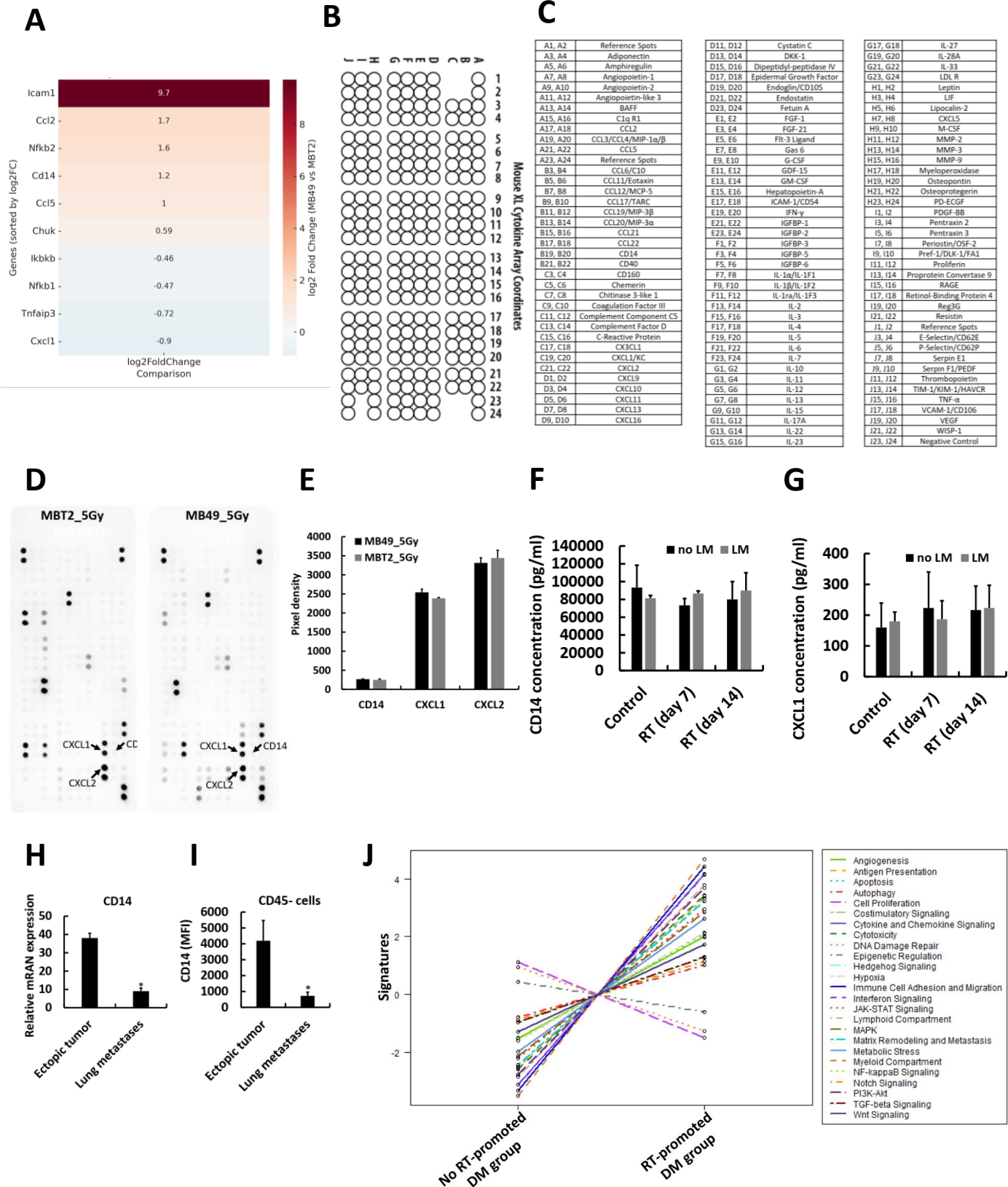

# Figure S6

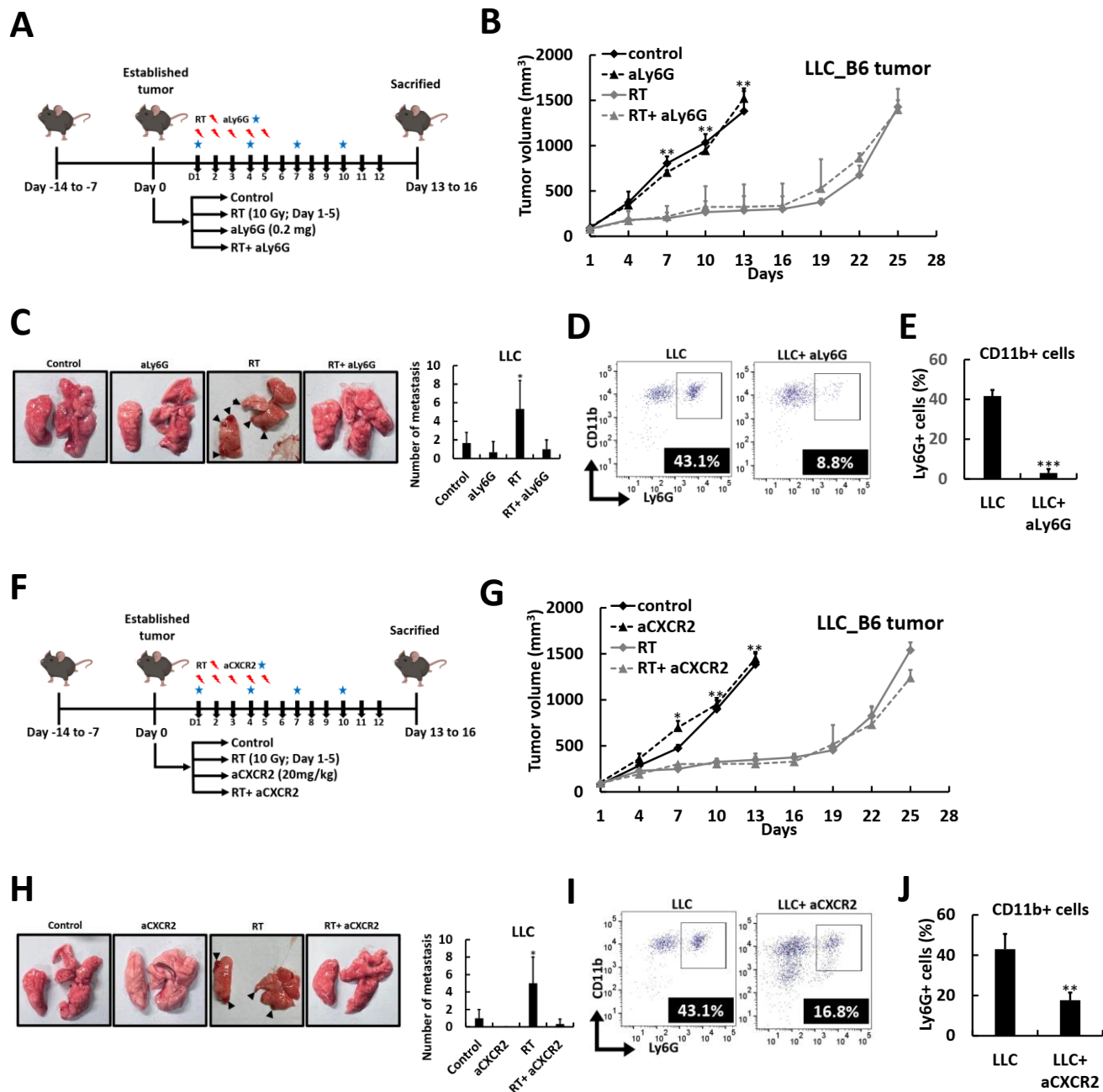

# Figure S7

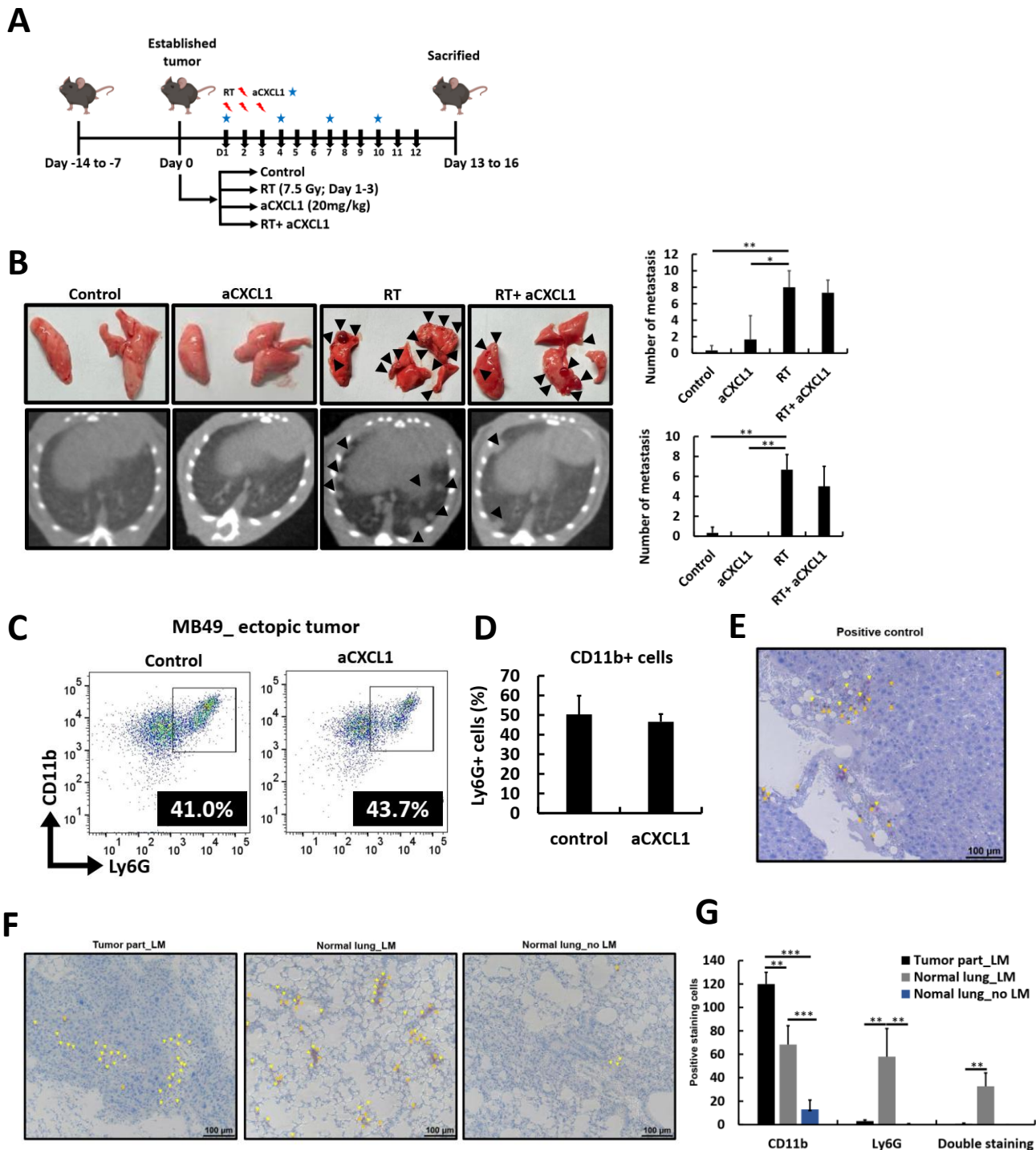

# Figure S8

**A**

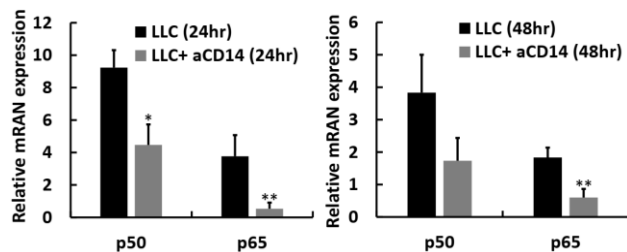

**B**

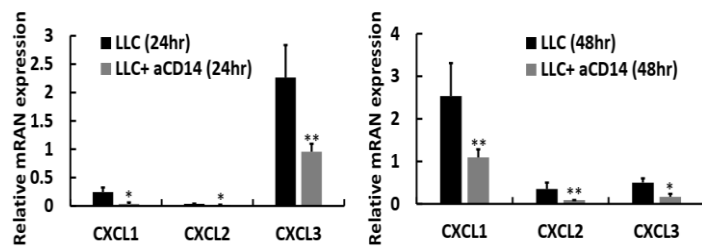

**C**

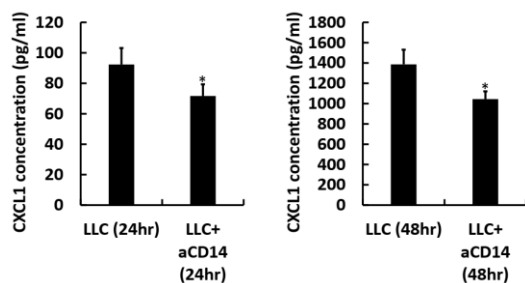

**D**

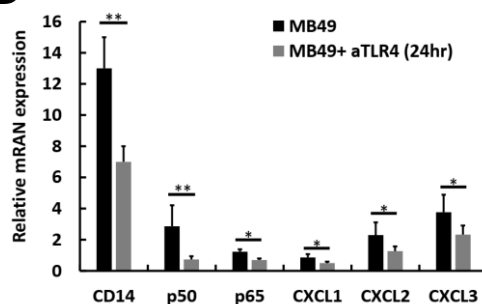

**E**

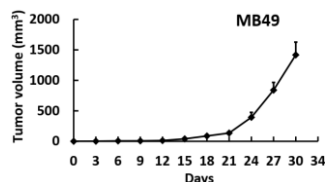

**F**

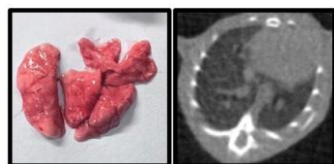

**G**

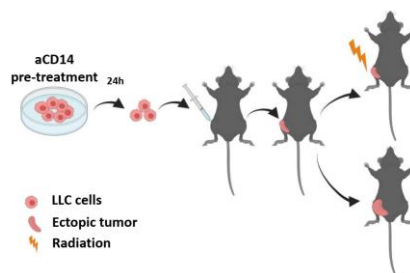

**H**

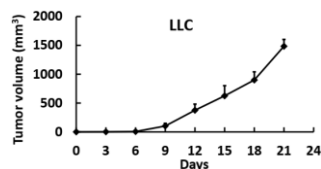

**I**

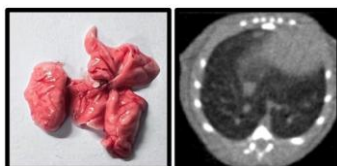

**J**

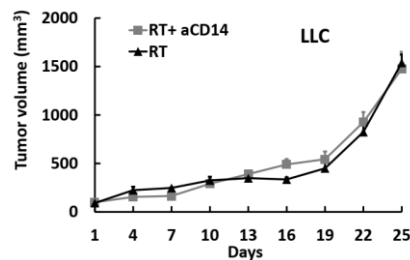

**K**

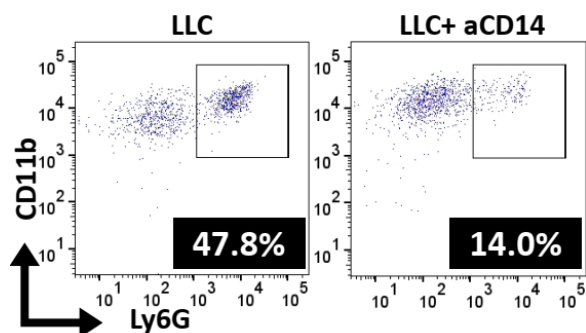

**L**

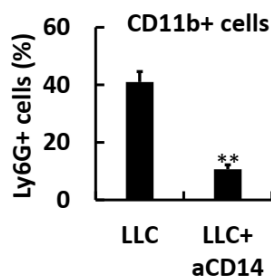

Supplement: Supplementary file 1 — Supplementary material 1. [file 12929_2025_1201_MOESM1_ESM.pdf]
