# Supplementary material for: High-CD14-expressing urothelial cancer cells foster a neutrophil-rich tumor microenvironment that increases the risk of radiation-promoted distant metastasis
Source: J Biomed Sci. 2026 Jan 4;33:2. doi: 10.1186/s12929-025-01201-2 (PMC12765301; doi:10.1186/s12929-025-01201-2)
Supplement: Supplementary file 2 — Supplementary material 2. [file 12929_2025_1201_MOESM2_ESM.docx]

**Figure S1.** Patient no. 2 in the radiotherapy (RT)-promoted distant metastasis (DM) group had residual paraaortic lymphadenopathy (LAP) after chemotherapy, indicated by an asterisk, as seen on both (**A**, left) the positron emission tomography (PET) scan and (**A**, right) the computed tomography (CT) scan. Two months after RT, the irradiated LAP remained stable (**B**); however, CT scans revealed multiple new liver metastases (**C**, left, asterisk) and lung metastases (**C**, right, asterisk). Patient no. 3 in the RT-promoted DM group had residual intraabdominal LAP (asterisk), as shown on the MR image (**D**). One month after RT, the irradiated LAP remained stable (**E**); however, MRI revealed multiple new liver metastases (**F,** asterisk). (**G**) Kaplan–Meier curves show overall survival in patients with CXCR2-positive tumors (≥5% of tumor cells) compared with CXCR2-negative tumors (<5% of tumor cells). Numbers at risk are indicated below the plot. (**H**)The neutrophil-to-lymphocyte ratio and (**I**) neutrophil percentage in the peripheral blood were compared between 24 patients in the RT-promoted DM group and 6 patients in the non-RT-promoted DM group. (**J**) A time course plot illustrates changes in the percentage of neutrophils in the peripheral blood before RT, within one month post-RT, and more than one month after RT.

**Figure S2.** (**A‒K**) Score for various immune cells generated using advanced analysis with nSolver analysis software.

**Figure S3.** (**A**) The in vivo experimental workflow involved the subcutaneous inoculation of C3H mice in their hind limbs with 2x10^6^ MBT2 bladder cancer cells. Once the tumors were established (day 0; mean starting tumor volume = 98 mm^3^), the mice were randomly assigned to the no radiotherapy (RT) group or the RT group. For the mice in the RT group, thigh tumors were irradiated with three 7.5-Gy fractions using a small animal X-ray irradiator. (**B**) The tumor growth curves revealed local control by RT. The data points represent the mean tumor volume from each group measured on the indicated day. (**C**) Representative images revealed no lung metastasis on the cone-beam computed tomography scan (right) or in the gross lung specimen (left). (**D**) Representative scratch wound healing images of MB49 and MBT2 cells at 0, 6, 24, 48, and 72 h after wounding. (**E**) Quantification of wound closure (%) over time in MB49 and MBT2 cells. (**F**) Representative images (upper) of transwell migration assays in MB49 and MBT2 cells with or without 5 Gy irradiation, and quantification of migrated cell numbers at 24 h (lower). (**G**) The gating strategy employed for analyzing neutrophils, defined as CD11b+/Ly6G+ cells expressing CXC motif chemokine receptor 2 (CXCR2) , as well as their polarization into N1 (ICAM1⁺) and N2 (CD206⁺) subsets, in the *in vivo* experiment. The values are presented as means ± SDs. **P* < 0.05; ***P* < 0.01. RT: radiotherapy.

**Figure S4.** (**A**) The gating strategy employed in the *in vivo* experiment to analyze neutrophils expressing CD18, as well as tumor cells expressing ICAM1 and CD14. Representative flow cytometry analyses show the consistent high CD18 expression levels on neutrophils within intrinsic ectopic tumors, as well as ectopic tumors at 7 days after radiotherapy (RT), in both (**B**) MB49 and (**C**) LLC mouse models. Representative flow cytometry analyses display the percentage of ICAM1 expression on tumor cells, identified as CD45- cells, alongside their quantified numbers within intrinsic ectopic tumors and in ectopic tumors at 7 days after RT in both (**D**) MB49 and (**E**) LLC mouse models. (**F**) Representative plots and quantification of CD11b⁺/Ly6G⁺ neutrophils in ectopic tumors versus lung metastases. (**G**) Flow cytometry showing CXCR2 expression on neutrophils from ectopic tumors and lung metastases, with corresponding quantification. (**H**) CD18 expression on neutrophils from ectopic tumors and lung metastases, presented as representative plots and mean fluorescence intensity (MFI). Flow cytometry showing neutrophil subsets with (**I**) representative plots of N1 (ICAM1⁺) and N2 (CD206⁺) neutrophils at control, RT day 7, and RT day 14, (**J**) quantification of N1 and N2 proportions, and (**K**) calculated N1/N2 ratios across time points. The values are presented as means ± SDs. **P* < 0.05.

**Figure S5.** (**A**) Heatmap of CD14 and NFκB pathway–related genes from RNA-seq data (MB49 vs. MBT2 cells). Color scale and numbers indicates log2 fold change (MB49 relative to MBT2). The cell culture supernatant was tested with (**B**) the Proteome Profiler Mouse XL Cytokine Array to detect multiple cytokines and the expression of multiple chemokines, growth factors, and other proteins in (**C**) the culture medium samples. (**D**) Cytokine array analysis of culture medium from the MB49 (right) and MBT2 (left) cells 24 h after 5 Gy irradiation, and (**E**) quantification of neutrophil-recruiting chemokines levels in the two cell lines. ELISA measurement of serum CD14 (**F**) and CXCL1 (**G**) concentrations in MB49 tumor–bearing mice, comparing groups that developed RT-induced lung metastases (LM) versus those without LM at baseline (control), day 7, and day 14 post-RT. Comparison of CD14 expression between ectopic tumors and LM, assessed by qRT‒PCR (**H**) and by flow cytometry in CD45⁻ cells that include tumor cells (**I**). (**J**) Signaling pathway scores were compared between the radiation (RT)-promoted distant metastasis (DM) group and the non-RT-promoted DM group using advanced analysis with nSolver software. The values are presented as means ± SDs. **P* < 0.05.

**Figure S6.** (**A**) The experimental workflow of the LLC ectopic mouse model is outlined; the treatment responses were compared among the control group, the radiotherapy (RT) group, the Ly6G antagonist (aLy6G) group, and the RT plus aLy6G group. Tumor growth curves (**B**) and the number of pulmonary metastases, as indicated by gross surface lesions (**C**), were compared. A representative flow cytometry figure demonstrating the CD11b+/Ly6G+ population is presented (**D**), along with the corresponding percentages (**E**) in LLC ectopic tumors from the control and aLy6G groups. (**F**) The experimental workflow was similar for the LLC ectopic mouse model; the treatment responses were compared among the control group, the RT group, the CXCR2 antagonist (aCXCR2) group, and the RT plus aCXCR2 group. Tumor growth curves (**G**) and the number of pulmonary metastases, as indicated by gross surface lesions (**H**), were compared. A representative flow cytometry figure showing the CD11b+/Ly6G+ population is presented (**I**), along with the corresponding percentages (**J**) in LLC ectopic tumors from the control and aCXCR2 groups. The values are presented as means ± SDs. **P* < 0.05; ***P* < 0.01. RT: radiotherapy; aLy6G: Ly6G antagonist; aCXCR2: CXCR2 antagonist.

**Figure S7.** (**A**) The experimental workflow of the MB49 ectopic mouse model is outlined; the treatment responses were compared among the control group, the radiotherapy (RT) group, the CXCL1 antagonist (aCXCL1) group, and the RT plus aCXCL1 group. The number of pulmonary metastases, as indicated by gross surface lesions (**B**, upper row; arrow) and on computed tomography (CT) (**B**, lower row; arrow), were compared. A representative flow cytometry figure demonstrating the CD11b+/Ly6G+ population is presented (**C**), along with the corresponding percentages (**D**) in MB49 ectopic tumors from the control and aCXCL1 groups. Representative double IHC staining for CD11b (red) and Ly6G (brown), with (**E**) B6 mouse spleen as a positive control showing CD11b⁺ (yellow arrowheads) and Ly6G⁺ (orange arrowheads) cells, (**F**) lung tissues including a metastatic lesion enriched in CD11b⁺ cells (left), adjacent normal lung from the same lung metastases (LM)-bearing mouse (middle), and normal lung from a mouse without metastasis (right) and (**G**) quantification of CD11b⁺ and Ly6G⁺ cells. Scale bars, 100 μm. The values are presented as means ± SDs. **P* < 0.05; ***P* < 0.01; ****P* < 0.001. RT: radiotherapy; aCXCL1: CXCL1 antagonist; LM: lung metastases.

**Figure S8.** Real-time quantitative reverse transcription (qRT–PCR) analysis of (**A**) genes associated with NFκB signaling and (**B**) chemokines involved in neutrophil recruitment in LLC cells pretreated with or without a CD14 antagonist (aCD14) for 24 h and 48 h. (**C**) Enzyme-linked immunosorbent assay of CXCL1, a neutrophil-recruiting chemokine, in LLC cells pretreated with or without aCD14 for 24 h or 48 h. (**D**) Relative mRNA expression of CD14, NFκB subunits (p50, p65), and neutrophil-recruiting chemokines (CXCL1, CXCL2, CXCL3) in MB49 cells treated with or without a TLR4 antagonist (aTLR4, 24 h), measured by qRT–PCR. (**E**) Tumor growth curves for ectopic tumors established from aCD14-pretreated MB49 cells; day 0 refers to the cell implantation day. (**F**) Representative images of gross lung specimens and CT scans of lungs without metastasis after RT to ectopic tumors established from aCD14-pretreated MB49 cells are shown. (**G**) In the experimental workflow, LLC cells were pretreated with or without aCD14 for 24 h to investigate the effects of CD14 suppression on radiotherapy (RT)-promoted lung metastasis and neutrophil infiltration in ectopic tumors. (**H**) Tumor growth curves for ectopic tumors established from aCD14-pretreated LLC cells; day 0 refers to the cell implantation day. (**I**) Representative images of gross lung specimens and CT scans of lungs without metastasis after RT to ectopic tumors established from aCD14-pretreated LLC cells are shown. (**J**) The growth curves for tumors from the RT groups generated from LLC cells pretreated with or without aCD14 are displayed, with day 0 referring to the day of tumor establishment (mean starting tumor volume = 98 mm^3^). A representative flow cytometry analysis demonstrates (**K**) the percentage of neutrophils in ectopic LLC tumors, alongside (**L**) their quantified numbers. The values are presented as means ± SDs. **P* < 0.05; ***P* < 0.01. RT: radiotherapy; aCD14: CD14 antagonist; aTLR4: TLR4 antagonist.
