# Supplementary material for: High-CD14-expressing urothelial cancer cells foster a neutrophil-rich tumor microenvironment that increases the risk of radiation-promoted distant metastasis
Source: J Biomed Sci. 2026 Jan 4;33:2. doi: 10.1186/s12929-025-01201-2 (PMC12765301; doi:10.1186/s12929-025-01201-2)
Supplement: Supplementary file 3 — Supplementary material 3. [file 12929_2025_1201_MOESM3_ESM.docx]

Supplementary Table 1. Clinical characteristics of 7 urothelial carcinoma patients included in the Nanostring analysis

|  | Age | Sex | KPS | Primary  cancer  type | Systemic  therapy  during RT | Systemic  therapy regimen | No. of ML before RT | Irradiated  tumor  location(s) | Irradiated tumor  volume (ml): CTV/PTV | RT  fractionation | New MLs within 3m after RT | Organs with  new ML  (No.) |
| --- | --- | --- | --- | --- | --- | --- | --- | --- | --- | --- | --- | --- |
| Patient 1 | 64 | M | 90 | Renal UC | Yes | Gemcitabine,  Cisplatin | 1 | PA LAP | 87/174 | 50 Gy/ 20 fx | Yes | Lung (12)  Liver (> 20) |
| Patient 2 | 41 | M | 90 | Bladder UC | Yes | Pembrolizumab | 2 | Pelvic LAP | 514/770 | 50 Gy/ 25 fx | Yes | Bone (3)  Liver (> 20)  Brain (3) |
| Patient 3 | 90 | M | 80 | Bladder UC | No | N.A. | 3 | PA/ Pelvic LAP | 747/1175 | 45 Gy/ 10 fx | Yes | Liver (15) |
| Patient 4 | 76 | M | 90 | UTUC | Yes | Pembrolizumab | 2 | RP LAP | 212/361 | 45 Gy/ 15 fx | No | N.A. |
| Patient 5 | 72 | M | 90 | Bladder UC | No | N.A. | 4 | PA LAP | 283/515 | 50 Gy/ 25 fx | No | N.A. |
| Patient 6 | 77 | F | 80 | UTUC | No | N.A. | 3 | PA/ Pelvic LAP | 726/1129 | 50 Gy/ 20 fx | No | N.A. |
| Patient 7 | 61 | M | 90 | Bladder UC | Yes | MBG453, PDR001 | 3 | PA LAP | 1152/1552 | 55 Gy/ 25 fx | No | N.A. |

KPS: Karnofsky performance scale; UC: urothelial carcinoma; UTUC: upper tract urothelial carcinoma; RT: radiotherapy; PA: para-aortic; LAP: lymphadenopathy; RP: retroperitoneal; fx: fraction; CTV: clinical target volume; PTV: planning target volume; ML: metastatic lesions; N.A.: not applicable.
